# Imaging *Staphylococcus aureus* biofilms *in vivo* reveals neutrophil exclusion by PNAG exopolysaccharide

**DOI:** 10.1128/mbio.00522-26

**Published:** 2026-07-31

**Authors:** Rachel M. Kratofil, Rehnuma T. Sejuty, Trevor E. Randall, Josefien W. Hommes, Jessica Chisholm, Michelle Willson, Raymond Shim, Deepa Raju, Mario A. Vargas, P. Lynne Howell, Gerald B. Pier, Douglas W. Morck, Joe J. Harrison, Paul Kubes

**Affiliations:** 1 Department of Physiology and Pharmacology, University of Calgary 198999, Calgary, Alberta, Canada; 2 Calvin, Phoebe, and Joan Snyder Institute for Chronic Diseases, University of Calgary 157743, Calgary, Alberta, Canada; 3 Department of Biological Sciences, University of Calgary 98634, Calgary, Alberta, Canada; 4 Department of Comparative Biology and Experimental Medicine, University of Calgary 2129, Calgary, Alberta, Canada; 5 Program in Molecular Medicine, The Hospital for Sick Children 7979, Toronto, Ontario, Canada; 6 Department of Biochemistry, University of Toronto 233836, Toronto, Ontario, Canada; 7 Division of Infectious Diseases, Department of Medicine, Brigham and Women’s Hospital/Harvard Medical School 370908, Boston, Massachusetts, USA; Universite de Geneve, Geneva, Switzerland

**Keywords:** neutrophils, biofilms, *Staphylococcus aureus*, video microscopy, exopolysaccharide

## Abstract

*Staphylococcus aureus* forms biofilms on biotic and abiotic surfaces, enabling persistent infections that evade immune clearance. Although many *S. aureus* strains can produce the biofilm-associated exopolysaccharide, poly-β-1,6-N-acetyl-D-glucosamine (PNAG), they often form protein-dominated biofilm matrices *in vitro*, leaving a role for PNAG unclear. Using intravital imaging in a foreign-body infection model, we found that PNAG in biofilms hinders neutrophil access and delays bacterial clearance. Neutrophil elastase was crucial for eventual biofilm clearance. *In vivo* PNAG labeling revealed that the exopolysaccharide forms a physical barrier that prevents neutrophils from reaching bacterial clusters. In contrast, PNAG-deficient strains permitted greater neutrophil infiltration and were cleared more rapidly than wild-type bacteria. Enzymatic degradation of PNAG with the glycoside hydrolases PgaB or dispersin B (DspB) disrupted the biofilm, restored neutrophil access, and enhanced bacterial clearance. Together, these findings identify PNAG as a key structural barrier protecting *S. aureus* from innate immunity and suggest that targeting PNAG with glycoside hydrolases may offer a promising therapeutic strategy for biofilm-associated *S. aureus* infections.

**IMPORTANCE:**

The biofilm-associated exopolysaccharide PNAG is frequently expressed in *Staphylococcus aureus* clinical isolates but is often reduced during laboratory passage, with expression highly dependent on growth conditions. While *in vitro* analyses have revealed that PNAG is not a dominant matrix component, our intravital imaging of community-acquired methicillin-resistant *S. aureus* (CA-MRSA) skin infections demonstrates that PNAG is robustly produced *in vivo* and plays a central role in immune evasion. These findings highlight how PNAG function in tissue environments may be non-obvious *in vitro* and underscore the need for *in vivo* models to understand biofilm pathogenesis. By revealing PNAG as a key barrier to neutrophil-mediated clearance, this work positions PNAG and PNAG-targeting glycoside hydrolases as compelling therapeutic candidates for treating antibiotic-resistant *S. aureus* biofilm infections, a major cause of morbidity in both healthcare and community settings.

## INTRODUCTION

In 2019, *Staphylococcus aureus* infections caused over one million deaths worldwide, with tens of thousands of deaths linked to skin and subcutaneous infections (1). Antimicrobial-resistant *S. aureus* contributed to an estimated 178,000 deaths (2), highlighting the urgent need for antibiotic alternatives. *S. aureus* biofilms on medical devices and host tissues like bone and heart valves contribute to chronic infections (3). These biofilms resist the immune system and antimicrobials, making them difficult to eradicate (4). Standard treatment involves removing infected devices, increasing the risk of complications (5). Currently, no approved therapies specifically target *S. aureus* biofilms.

A defining feature of biofilms, extensively characterized through *in vitro* studies, is their extracellular matrix (ECM). This matrix contains a diverse array of molecules, such as polysaccharides, proteins, teichoic acids, and extracellular DNA (eDNA), that together confer the structural and functional properties of the biofilm (3, 6). Early models categorized *S. aureus* biofilms based on ECM composition; however, emerging evidence suggests that biofilm architecture is highly dynamic, with the molecular makeup changing over time and across spatial niches as the biofilm matures (7). Although the specific composition varies by strain, early-stage *S. aureus* biofilms are typically enriched in eDNA and a repertoire of cell wall-anchored proteins known as microbial surface components recognizing adhesive matrix molecules (MSCRAMMs), many of which are bona fide virulence factors (8). As biofilms develop, their ECM becomes further enriched with eDNA and amphipathic α-helical peptides termed phenol-soluble modulins (PSMs) that are highly pro-inflammatory (7, 9). Notably, recent *in vivo* studies have revealed that bacterial eDNA contributes to biofilm-like lesion formation in the kidney, where it promotes neutrophil exclusion and immune evasion (10). Collectively, these findings, supported by both *in vitro* and *in vivo* studies using advanced molecular and imaging approaches, have led to the prevailing view that proteins and eDNA represent dominant structural and functional components of the *S. aureus* biofilm ECM.

Another key ECM component in Staphylococcal species is poly-β-1,6-N-acetyl-D-glucosamine (PNAG), originally identified in *Staphylococcus epidermidis* as the polysaccharide intercellular adhesin (PIA) (11). PNAG consists of repeating monomers of β-1-6-linked N-acetyl glucosamine and is synthesized by the enzymatic machinery encoded by the *icaADBC* operon (12, 13). *IcaB* partially deacetylates PNAG, altering its charge to enable stable biofilm formation (14, 15). In *S. epidermidis*, PNAG is critical for catheter-associated infections *in vivo*, as *ica-*deficient strains exhibit faster bacterial clearance than their wild-type counterparts (16). Importantly, PNAG production is tightly controlled by a layered network of transcriptional regulators, including the repressors IcaR and Rob, both of which independently suppress *icaADBC* transcription. In a recent study, loss-of-function mutations in either *icaR* or *rob* led to increased biofilm elaboration and PNAG synthesis, and double mutations of *icaR* and *rob* are epistatic, resulting in a hyper-biofilm phenotype (17).

While much of the foundational work on PNAG has been conducted in *S. epidermidis*, the *icaADBC* operon has also been identified in multiple *S. aureus* strains, including MW2. In *S. aureus*, PNAG appears to be produced during the early stages of biofilm development (18); however, its contribution to biofilm stability is often less pronounced in *in vitro* model systems (18–21). For example, *S. aureus* strains such as UAMS-1 and USA300 JE2, harboring engineered loss-of-function mutations in the *ica* operon, still form early biofilms comparable to their isogenic wild-type counterparts (18–21). Despite this, several *in vivo* studies indicate that PNAG plays a measurable role in *S. aureus* pathogenesis. In a murine bacteremia model, PNAG contributed to bacterial virulence and lethality (22), and similar findings were observed in lung infection models, where *ica-*deficient strains displayed diminished bacterial burden and reduced inflammation (23). Additional *in vivo* work using systemic infection models has shown that deletion of the *icaADBC* operon reduces bacterial dissemination to secondary organs, including the kidneys, and protects mice from lethal infection (22). Together, these studies underscore that PNAG contributes to *S. aureus* virulence across multiple *in vivo* contexts; however, the precise host mechanisms by which PNAG-mediated biofilms promote immune evasion or persistence remain largely unexplored.

In this study, we used a foreign body biofilm infection model using *S. aureus*-embedded agar beads to visualize the host response to *S. aureus* biofilm. We found that bacterial persistence depended on PNAG in the biofilm matrix, which served as a physical barrier preventing neutrophil access. Infection with PNAG-deficient *S. aureus* increased immune cell infiltration at 24 h and promoted bacterial clearance by 7 days. Intravital imaging showed that neutrophils used elastase to penetrate the infection site but remained separated from bacterial clusters by PNAG. In contrast, infection with PNAG-deficient strains allowed neutrophils to reach and clear bacteria. Finally, enzymatic degradation of PNAG using PgaB, which hydrolyzes partially deacetylated PNAG (24), enhanced neutrophil access and eradicated infection *in vivo*. Analogous results were observed for dispersin B (DspB). Together, these findings demonstrate that PNAG impedes innate immune clearance and that targeting PNAG may provide a therapeutic strategy against *S. aureus* biofilm infections.

## RESULTS

### PNAG contributes to *S. aureus* MW2 biofilm formation *in vitro*

**Fig 1:**
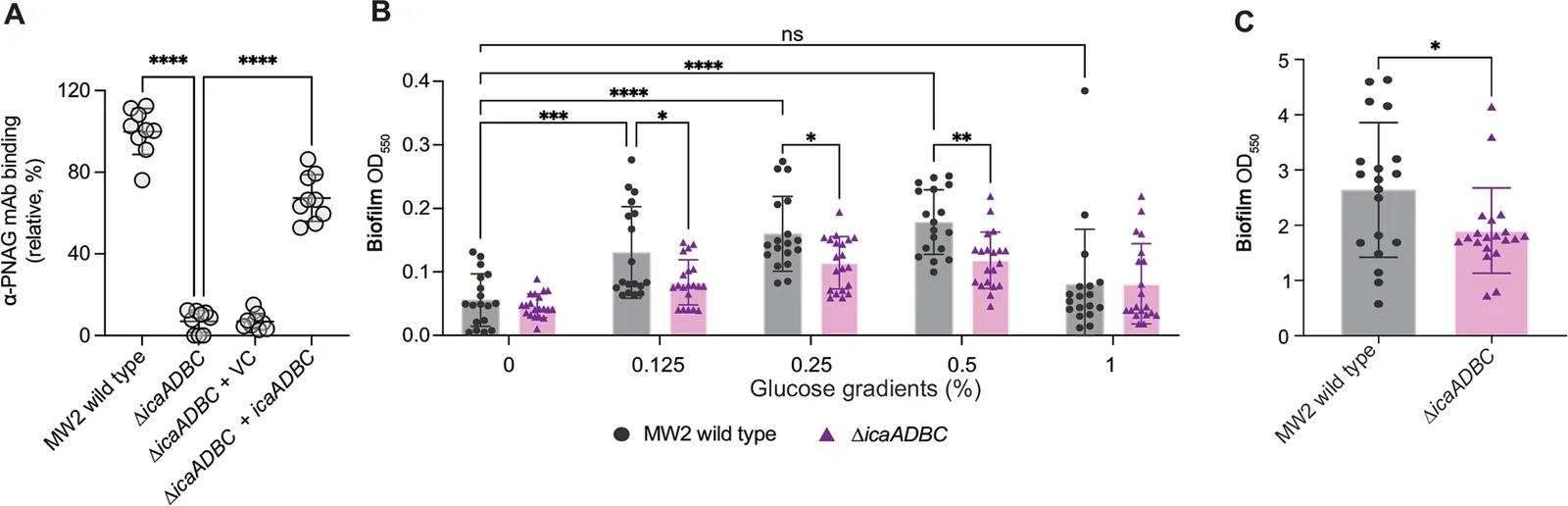
PNAG expression and *in vitro* validation of Δ*icaADBC* mutants. (**A**) Quantification of semi-quantitative PNAG dot blot. (**B**) Quantification of *in vitro* biofilm production using the Calgary Biofilm Device with TSB containing increasing glucose gradients. (**C**) Quantification of *in vitro* biofilm production using the O’Toole microplate assay in TSB containing 0.125% glucose. *P < 0.05, **P < 0.01, ***P < 0.001, ****P < 0.0001. One-way ANOVA with Tukey’s *post-hoc* test (**A**), Mixed-effects analysis with Šídák’s multiple comparisons test (**B**), and Student *t*-test (**C**) were used. VC: vector control.

The *icaADBC* operon encodes enzymatic machinery responsible for PNAG synthesis (13). To investigate PNAG-deficient phenotypes, we examined biofilm formation in *S. aureus* MW2 and an isogenic, genome-edited mutant lacking the *ica* operon (Δ*icaADBC*) (25). Deletion of this locus abolished PNAG production, as confirmed by dot blot analysis using a PNAG-specific, human IgG1 monoclonal antibody (hereafter referred to as anti-PNAG, Fig. 1A). Similar results showing loss of PNAG production were observed with isogenic Δ*icaA* and Δ*icaC* strains (Fig. S1A). Genetic complementation of the Δ*icaADBC* strain with *icaADBC* expressed *in trans* rescued PNAG expression in the Δ*icaADBC* strain (Fig. 1A), validating the specificity of the anti-PNAG monoclonal antibody for PNAG and confirming the genetic linkage between PNAG production and the *icaADBC* locus in the mutant strain.

Consistent with prior reports that *S. aureus* biofilm formation is affected by glucose (26), we observed that biofilm formation by the wild-type strain was enhanced in media supplemented with glucose (Fig. 1B). We observed that the Δ*icaADBC* mutant developed significantly less biofilm than the wild-type strain in both the Calgary Biofilm Device (MBEC Assay) (Fig. 1B) and microplate crystal violet assays in media supplemented with 0.125% (wt/vol) glucose (Fig. 1C) (27–29). Detection of *ica-*dependent biofilm phenotypes *in vitro* required precise glucose concentrations; reduced biofilm biomass in *ica* mutants was only apparent within a narrow concentration range (0.125–0.5% [wt/vol]; 6.9–28 mM) and was lost at both extremes—in glucose-free media and at higher concentrations (1% [wt/vol]) (Fig. 1B). We posit that this result may be physiologically relevant to murine infection models because fasting glucose levels in mice are ~8.6–9.7 mM in serum (30), with these levels subject to post-prandial elevations. Nevertheless, these data demonstrate that PNAG contributes to *S. aureus* MW2 biofilm accumulation in a glucose-dependent manner and that *ica-*dependent phenotypes may be overlooked *in vitro* if growth conditions are not carefully controlled.

### PNAG is expressed by many laboratory strains and clinical isolates

Prior reports suggest that clinical isolates expressing *icaADBC* when first isolated may lose PNAG production with *in vitro* passage (31). Therefore, we evaluated PNAG production in a screen of 48 recently isolated, randomly selected *S. aureus* clinical isolates. We observed that all isolates were susceptible to opsonic killing mediated by anti-PNAG in the presence of human polymorphonuclear neutrophils and complement (Fig. S1B) (32). This result suggests that PNAG expression is widespread among clinical isolates.

To directly assess *ica-*dependent PNAG production in multiple genetic backgrounds, we generated Δ*icaADBC* mutants and complemented derivatives in *S. aureus* Newman, 10833, MN8, and USA300 LAC. Semi-quantitative dot blots showed that anti-PNAG reactive material was substantially reduced in all Δ*icaADBC* mutants and restored upon complementation (Fig. S1C).

While the *S. aureus* MW2 Δ*icaADBC* strain exhibited little background signal in this dot blot assay (Fig. 1A), we noticed that several other strain backgrounds exhibited elevated background signals (Fig. S1C). We hypothesized that this phenomenon was likely attributable to non-specific IgG binding via the surface proteins protein A (Spa) and/or Sbi (33). To directly address this hypothesis, we generated a Δ*spa* Δ*sbi* Δ*icaADBC* triple mutant in *S. aureus* Newman and repeated the anti-PNAG mAb binding assay. Deletion of *spa* and *sbi* alone significantly reduced the background signal compared to wild-type Newman, suggesting that protein A and Sbi may be drivers of non-specific antibody binding in this assay (Fig. S1D). By contrast, the Δ*spa* Δ*sbi* Δ*icaADBC* showed near-complete abolishment of background signal, confirming that residual signal in the Newman Δ*icaADBC* single mutant reflects non-specific IgG capture rather than PNAG-independent polysaccharide production (Fig. S1D). Taken together, these data confirm that PNAG production is *ica-*dependent across diverse laboratory and clinical strain backgrounds while also highlighting the importance of accounting for Spa- and Sbi-mediated background when interpreting dot blot assays in strains with high IgG-binding capacity.

### PNAG is expressed by *S. aureus* MW2 biofilms *in vivo*

**Fig 2:**
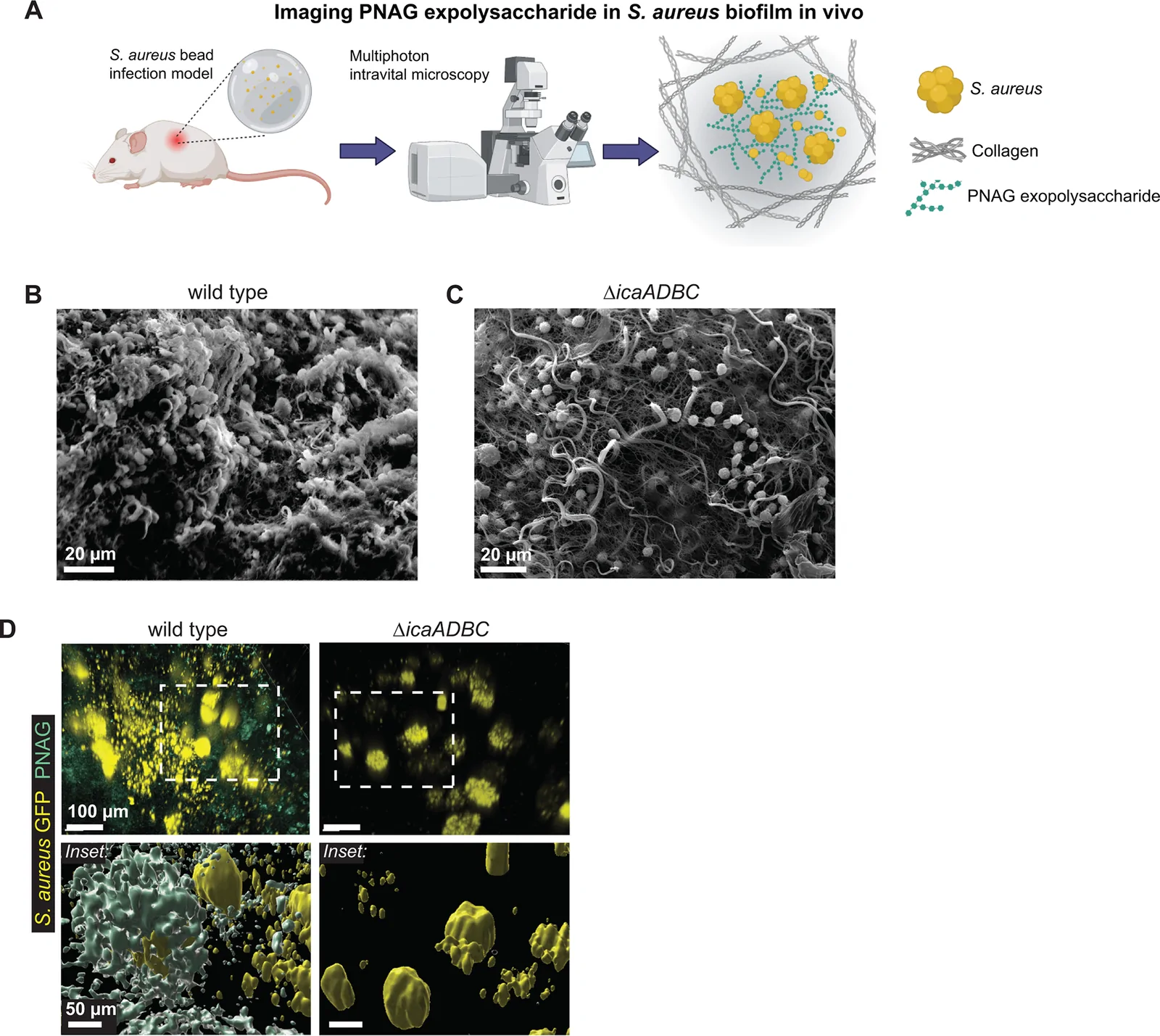
*S. aureus* forms PNAG-dependent biofilms *in vivo*. (**A**) Schematic of the *S. aureus* bead infection model and multiphoton intravital microscopy to image the biofilm infection *in vivo*. (**B and C**) Representative scanning electron microscopy images of MW2 wild type (**B**) and MW2 Δ*icaADBC S. aureus* bead (**C**) at 24 h post-infection. (**D**) C57 mice were infected with GFP-expressing MW2 wild type or Δ*icaADBC* and imaged at 24 h. Topical addition of Alexa Fluor 594-conjugated anti-F598 was added onto the skin infection prior to imaging. The image shows a 3D reconstruction (top) and surface rendering (bottom) of PNAG in wild-type and Δ*icaADBC* infections. Scale bars = 100 µm (top) and 50 μm (bottom).

We next visualized PNAG production during *S. aureus* MW2 biofilm infection *in vivo* using a foreign body agar bead model (Fig. 2A). In this model, bacteria are embedded within agar beads (500 CFU/bead), with the bead facilitating a persistent biofilm infection in the subcutaneous fascia of the dorsal skin as previously described (34). At 24 h post-infection, beads containing MW2 wild-type *S. aureus* formed interconnected clusters of bacteria surrounded by filamentous extracellular material consistent with a biofilm matrix (Fig. 2B). In contrast, beads containing the MW2 Δ*icaADBC* mutant displayed dispersed, individual bacteria without visible matrix connections (Fig. 2C).

To directly observe bacterial organization within intact tissue, we employed multiphoton intravital microscopy to enable real-time imaging of biofilm architecture and host responses in living mice. We employed a fluorescently conjugated anti-PNAG antibody to visualize PNAG localization within the infection site. Three-dimensional reconstructions revealed PNAG (cyan) surrounding wild-type bacterial clusters (yellow), whereas no anti-PNAG signal was detected in Δ*icaADBC* mutant infections (Fig. 2D ; Fig. S1E). These findings confirm that PNAG is produced by *S. aureus* biofilms *in vivo*.

### PNAG promotes bacterial persistence and impedes clearance

**Fig 3:**
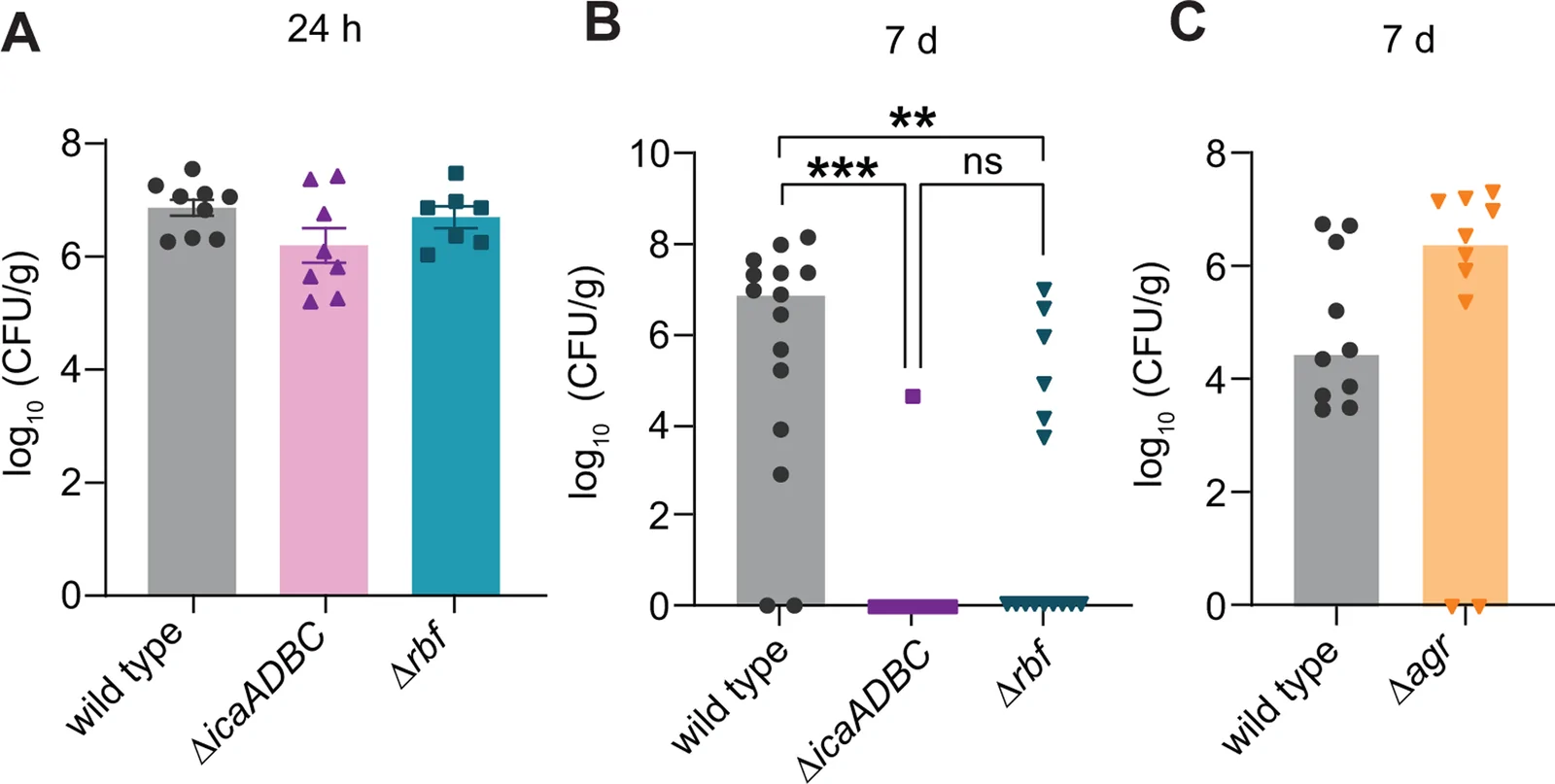
PNAG biofilm matrix contributes to bacterial persistence *in vivo*. (**A–C**) C57 mice were infected with *S. aureus* beads, and infections were harvested for the quantification of skin CFUs. (**A**) Bacterial CFUs at 24 h post-infection with MW2 wild-type, Δ*rbf,* and Δ*icaADBC*. *n* = 7–9 from two independent experiments. (**B**) Bacterial CFUs at 7 days post-infection with MW2 wild-type, Δ*rbf,* and Δ*icaADBC*. *n* = 10–15 from three independent experiments. (**C**) Bacterial CFUs at 7 days post-infection with MW2 wild type and Δ*agr*. *n* = 8–10 from two independent experiments. Kruskal-Wallis test (*P* = 0.0002) with Dunn’s multiple comparisons test (**B**) was used. **P < 0.01, ***P < 0.001.

To assess the functional consequence of PNAG expression in biofilms, we compared bacterial clearance in wild-type and PNAG-deficient infections. At 24 h post-infection, skin bacterial loads were similar between wild-type, Δ*icaADBC,* and Δ*rbf* (encoding a known positive regulator of the *icaADBC* operon (35)) (Fig. 3A). However, by 7 days post-infection, both Δ*icaADBC* and Δ*rbf* infections were largely cleared, whereas wild-type infections persisted in all mice (Fig. 3B). In contrast, the Δ*agr* mutant, which accumulates excess biofilm due to impaired quorum sensing and dispersal (36), remained detectable at 7 days (Fig. 3C). Notably, bacteria were not recovered from peripheral organs, including liver, spleen, lung, kidney, or blood, suggesting that the absence of biofilm did not increase bacterial dissemination (Fig. S2A). These results indicate that PNAG contributes to bacterial persistence by protecting biofilm-associated bacteria from host clearance mechanisms. Additionally, to test whether PNAG influences virulence during systemic infection, mice were challenged intravenously with MW2 wild-type, Δ*icaADBC,* and Δ*rbf* strains. Loss of PNAG did not affect survival or body weight (Fig. S2B and C). These findings suggest that PNAG’s primary role in promoting *S. aureus* MW2 persistence occurs locally at the biofilm infection site rather than during systemic dissemination.

### PNAG restricts neutrophil infiltration into the biofilm infection site

**Fig 4:**
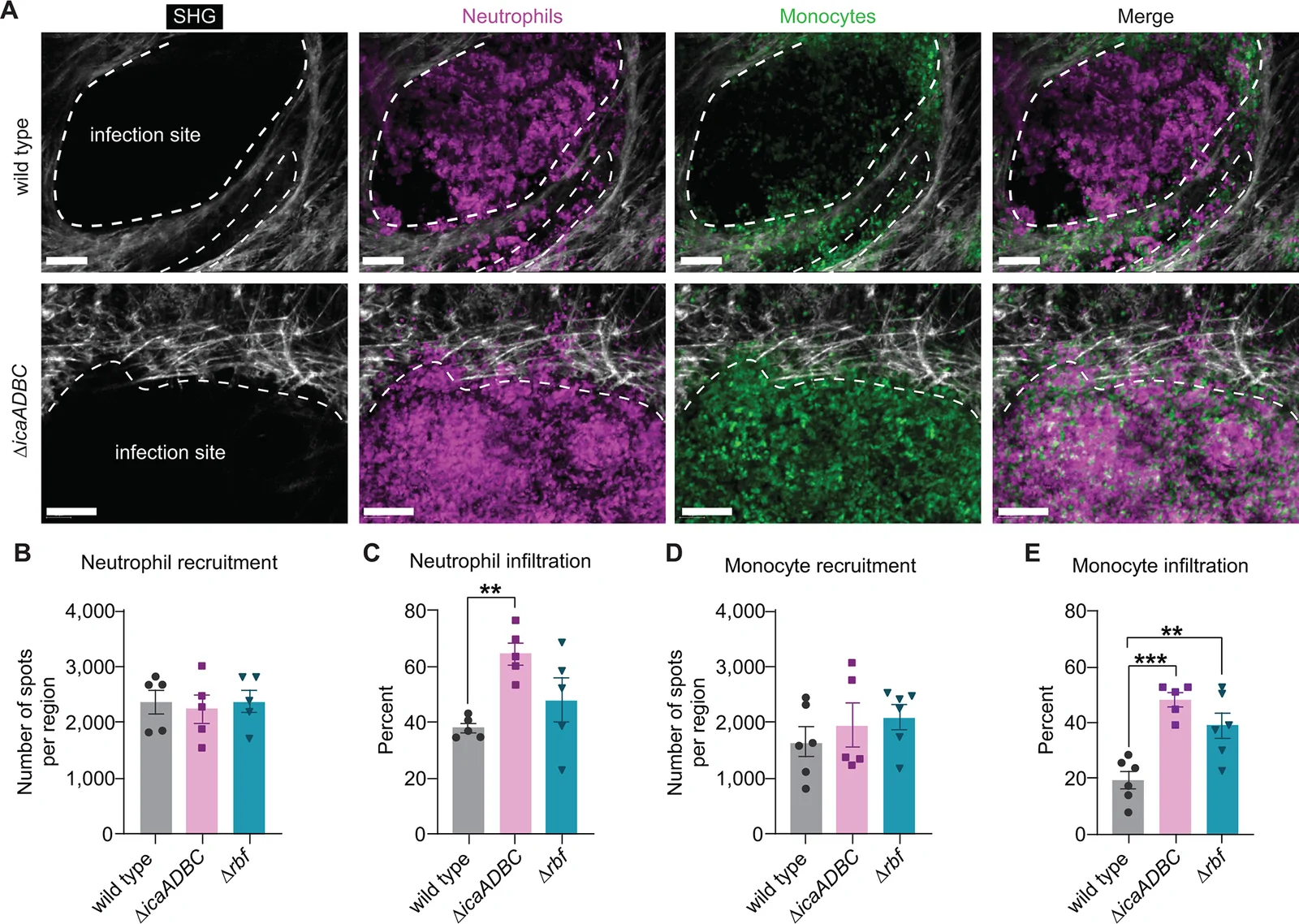
*S. aureus* biofilm blocks immune infiltration into the infection. (**A–E**) Catchup^ivm-red^ CX3CR1^gfp/wt^ mice were infected with *S. aureus* MW2 wild-type, Δ*rbf*, and Δ*icaADBC* beads and imaged at 24 h post-infection. (**A**) Representative stitched image of infections in wild-type and Δ*icaADBC*-infected mice. Scale bars = 100 µm. (**B–E**) Quantification of total numbers of neutrophils (**B**), percent neutrophil infiltration (**C**), total numbers of monocytes (**D**), and percent monocyte infiltration (**E**) at 24 h post-infection. *n* = 4 or 5 per group from two independent experiments. (**B–E**) One-way ANOVA (*P* = 0.0118 for C, *P* = 0.0003 for panel E) with Tukey’s multiple comparison test (**C and E**) was used. *P < 0.05, **P < 0.01, ***P < 0.001.

To investigate how PNAG influences immune cell infiltration, we built upon our previous work using intravital microscopy of *S. aureus* skin infection, which established how collagen architecture visualized by second-harmonic generation (SHG) can be used to accurately delineate the infection site *in vivo* (34). Using this imaging framework, we defined the infection zone as the SHG-negative region within the subcutaneous fascia and quantified immune cell infiltration by 3D image analysis (Fig. 4A). Although wild-type and Δ*icaADBC S. aureus* beads recruited similar total immune cell numbers (Fig. 4B and D), their spatial distribution differed. Only ~40% of neutrophils entered the SHG-negative core in wild-type infections, whereas infiltration increased significantly in Δ*icaADBC* infections (Fig. 4C). Monocyte entry showed a similar pattern (Fig. 4E), suggesting that PNAG broadly impedes leukocyte access rather than selectively affecting neutrophils. Flow-cytometric analysis confirmed comparable total neutrophil and monocyte numbers across all infection groups (Fig. S3A through C) and no major differences in neutrophil activation phenotype (Fig. S3D through F).

A key enzyme in neutrophils that is used to degrade ECM components is neutrophil elastase (37). We wondered whether neutrophil elastase would help neutrophils move into the infection core to clear bacteria. Indeed, neutrophil elastase-deficient mice infected with wild-type bacteria exhibited markedly reduced infiltration compared with C57BL/6 controls (Fig. S4A and B), indicating that neutrophil elastase facilitates neutrophil entry into the infection site.

**Fig 5:**
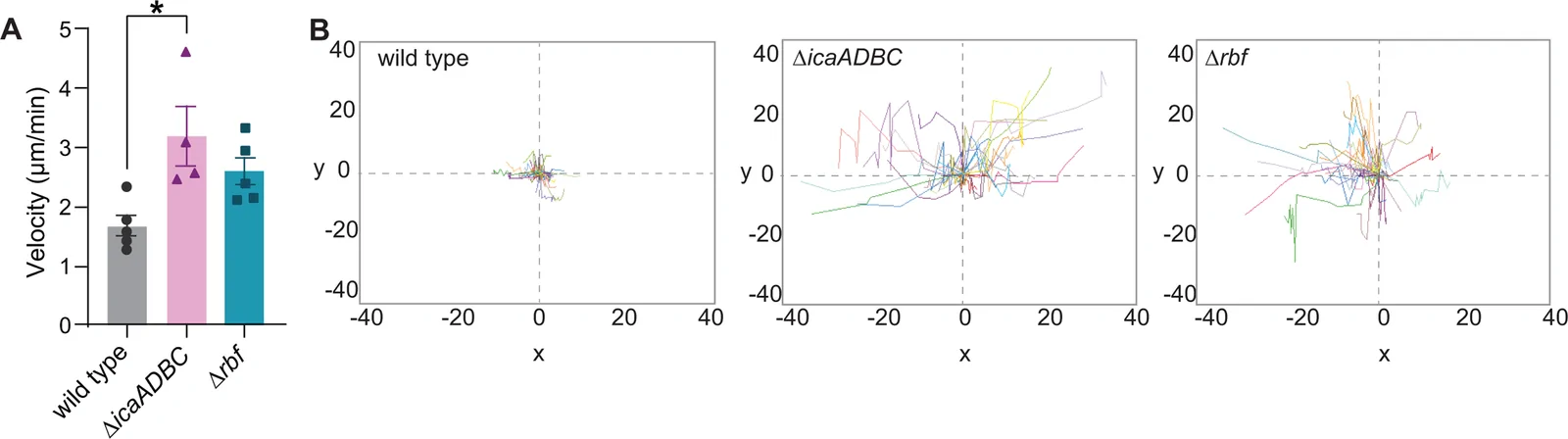
Neutrophil behavior during *in vivo* biofilm infection. (**A and B**) Catchup^ivm-red^ mice were infected with wild-type, Δ*rbf*, or Δ*icaADBC S. aureus* beads, and neutrophil behavior was analyzed at 24 h. (**A**) Quantification of neutrophil velocity over a 10-min video. *n* = 4 or 5 from two independent experiments. (**B**) Representative spider plots showing neutrophil displacement. Each neutrophil track is identified by a different color. One-way ANOVA with Tukey’s multiple comparison test (**A**) was used. *P < 0.05.

Intravital imaging at 24 h post-infection revealed that neutrophils in Δ*icaADBC* infections migrated with greater velocity and covered longer distances (20–40 μm) than those in wild-type infections (<20 µm) (Fig. 5A and B ; Video S1). These findings indicate that PNAG restricts neutrophil motility within the infected tissue.

**Fig 6:**
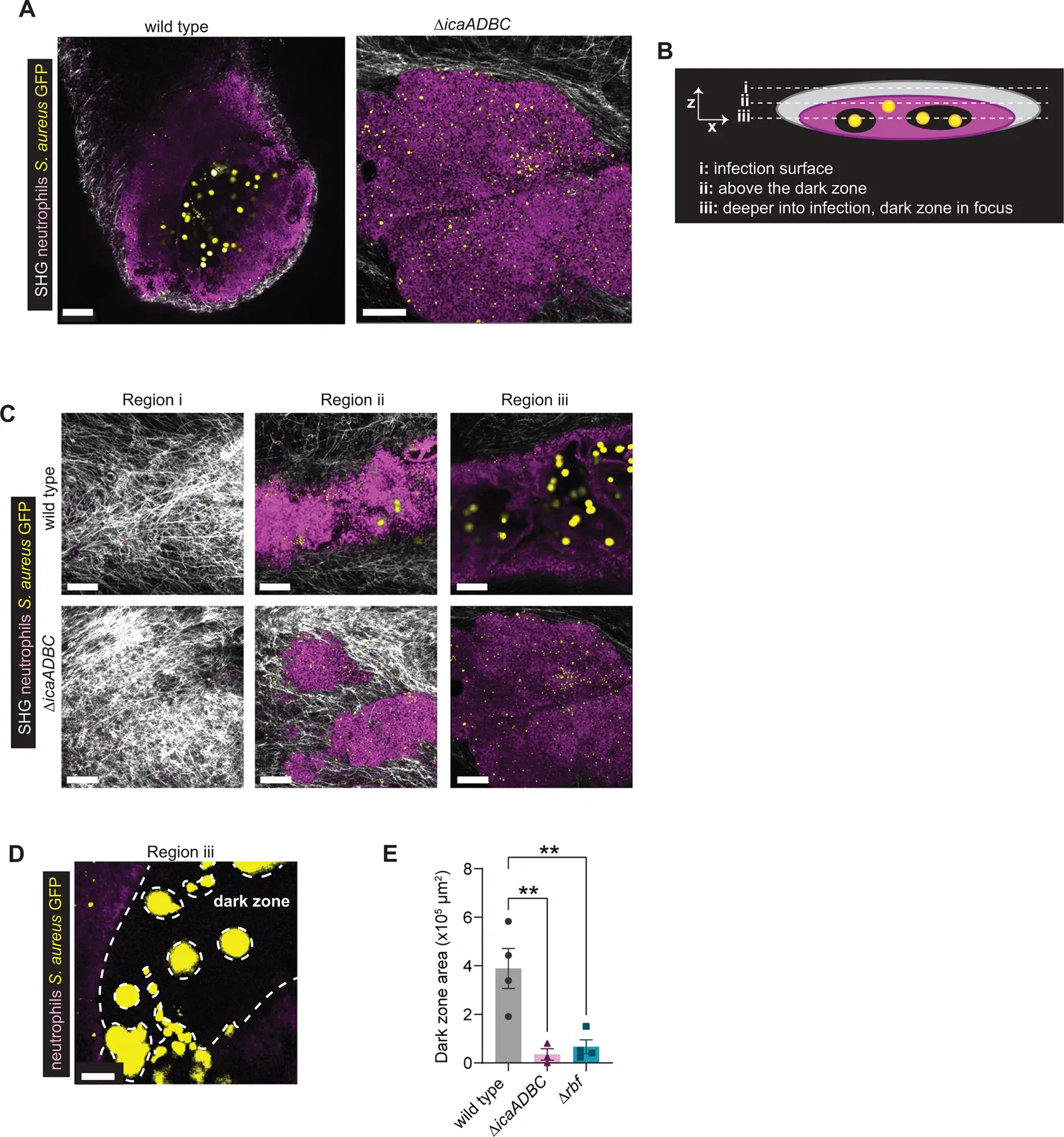
A dark zone defines the *S. aureus* PNAG biofilm matrix. (**A–E**) Catchup^ivm-red^ mice were infected with GFP-expressing *S. aureus* MW2 wild type, Δ*rbf*, and Δ*icaADBC* and imaged at 24 h. (**A**) Representative 2D images of wild-type and Δ*icaADBC* infections. Scale bar = 200 µm. (**B**) Schematic showing three focal planes of a 3D z-stack of the infection. (**C**) Representative images showing three focal planes of a 3D z-stack in wild-type and Δ*icaADBC* infections. Scale bar = 200 µm. (**D**) Representative image showing the dark zone highlighted by a dashed line. Scale bar = 50 µm. (**E**) Quantification of dark zone area in wild-type, Δ*rbf*, and Δ*icaADBC* infections. *n* = 3 or 4 per group from two independent experiments. (**E**) One-way ANOVA with Tukey’s multiple comparison test was used. **P < 0.01.

We next examined the spatial relationship between neutrophils and PNAG matrix and hypothesized that PNAG hindered neutrophil migration into the infection site. In Catchup^IVM-red^ mice infected with GFP-expressing *S. aureus*, a SHG-, GFP-, and tdTomato-negative region, herein termed the “dark zone,” was consistently observed surrounding wild-type bacterial clusters (Fig. 6A through D). This dark zone corresponded to PNAG-rich matrix visualized earlier with the anti-PNAG antibody (Fig. 2D) and was markedly reduced in Δ*rbf* and Δ*icaADBC* infections (Fig. 6E). To confirm that this phenotype was not strain-specific, we repeated the experiment using the clinical isolate *S. aureus* Newman, generating an isogenic Δ*icaADBC* knockout, and observed a similar loss of the dark zone phenotype in Newman Δ*icaADBC* infections (Fig. S5A and B). To further characterize the differences in ECM architecture across strains, we quantified bacterial aggregate number and size. Wild-type bacteria formed fewer but larger aggregates (53–94 aggregates per image; median area 24.28–108.63 µm²), whereas Δ*rbf* and Δ*icaADBC* strains produced smaller aggregates (Δ*rbf*: 253–6,770 aggregates per image; median area 2.40–17.30 µm²; Δ*icaADBC:* 12–1,103 per image; and median area 1.92–14.66 µm²) (Fig. S5C). These findings are consistent with PNAG, contributing to aggregate consolidation within the biofilm ECM. Taken together, these data suggest that PNAG forms a structural barrier that limits leukocyte access to bacteria *in vivo*.

### Enzymatic PNAG degradation restores immune access and improves bacterial clearance

**Fig 7:**
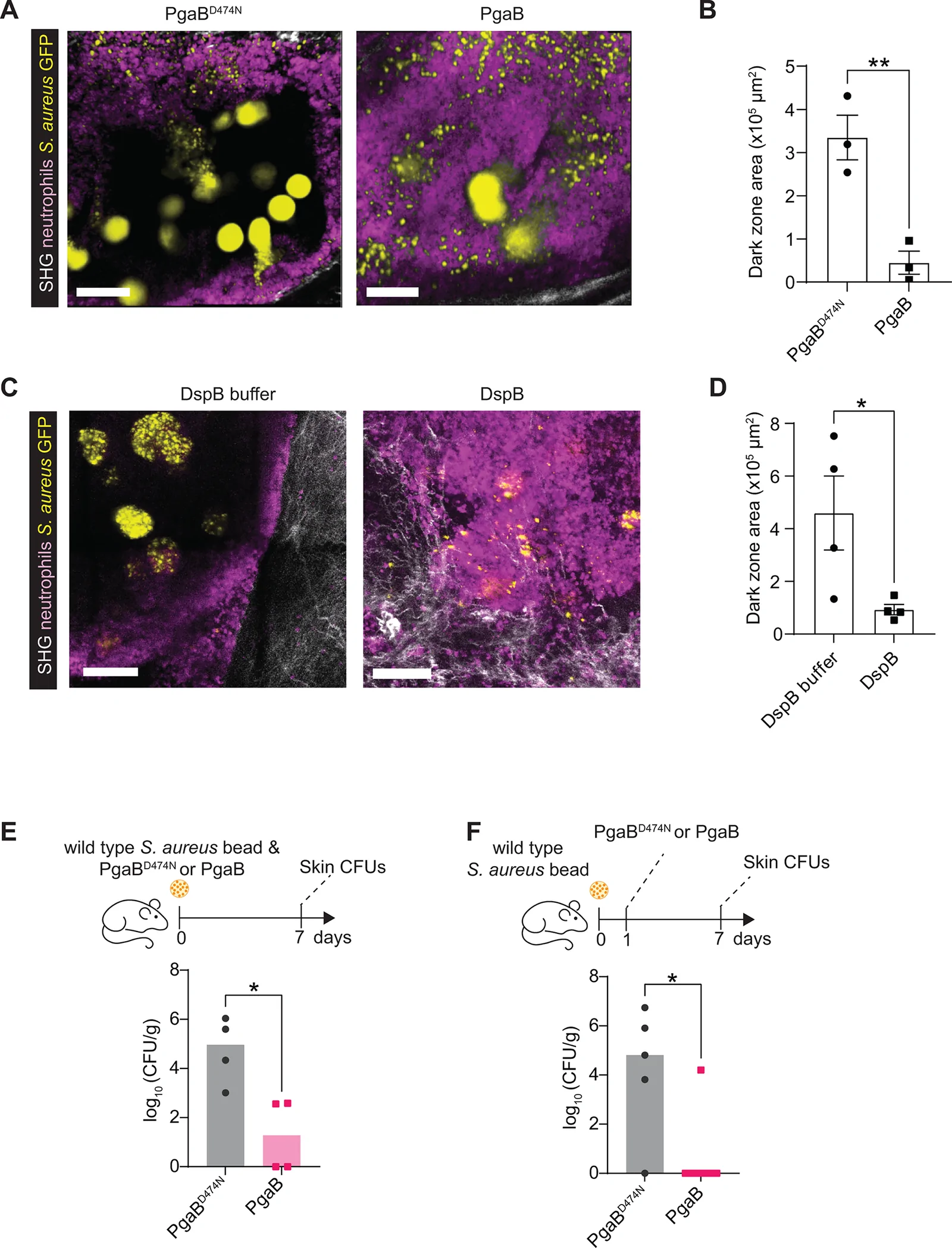
Glycoside hydrolase treatment disrupts *in vivo* biofilms. Catchup^ivm-red^ mice were infected with GFP-expressing *S. aureus* wild type, treated with PgaB or PgaB^D474N^, and imaged at 24 h. (**A**) Representative 2D images showing the dark zone after PgaB enzyme treatment. Scale bar = 100 µm. (**B**) Quantification of dark zone area in PgaB^D474N^- or PgaB-treated mice. *n* = 3 per group from two independent experiments. (**C**) Catchup^ivm-red^ mice were infected with GFP-expressing *S. aureus* wild type, treated with DspB or DspB buffer, and imaged at 24 h. Scale bar = 100 µm. (**D**) Quantification of dark zone area in DspB- or DspB buffer-treated mice. *n* = 4 per group from two independent experiments. (**E and F**) C57 mice were infected with *S. aureus* wild-type bead, treated with PgaB or PgaB^D474N^ at the time of infection (**E**) or at 24 h post-infection (**F**), and skin infections were harvested at 7 days post-infection for CFUs. *n* = 4 per group (**E**) and *n* = 5–7 per group (**F**) from two independent experiments. (**B, D, E, and F**) Student *t*-test was used. *P < 0.05, **P < 0.01.

Because PNAG-specific glycoside hydrolases such as PgaB and Dispersin B (DspB) can disrupt biofilms *in vitro* (24), we tested whether enzymatic treatment could overcome the PNAG barrier *in vivo*. Catchup^IVM-red^ mice infected with GFP-expressing wild-type *S. aureus* were treated with either active PgaB or inactive PgaB^D474N^ at infection onset. The dark zone persisted in control-treated mice but disappeared in PgaB-treated animals (Fig. 7A and B). Similarly, treatment with DspB reduced dark-zone area and enabled neutrophil contact with bacterial clusters (Fig. 7A and C). Both prophylactic (0 h) and therapeutic (24 h) PgaB treatments significantly lowered bacterial burden by day 7 (Fig. 7D and E), demonstrating that enzymatic PNAG degradation restores immune access and promotes clearance.

## DISCUSSION

### PNAG is linked to the persistence of *S. aureus* biofilms *in vivo*

Our data demonstrate that PNAG is critical for *S. aureus* persistence in a foreign-body subcutaneous infection model. Using intravital microscopy, we show that PNAG impedes immune infiltration into the infection, an effect not readily captured by conventional *in vitro* systems. These findings have implications for the development of experimental therapeutics targeting biofilm-associated infections.

We used our previously established agar bead model (34), originally used to study neutrophil responses at higher infectious doses (38), and employing a lower inoculum (500 CFU/bead). This modification better reflects early stages of infection, resulting in robust biofilm formation with a less overwhelming inflammatory response (34). These conditions may be more physiologically and clinically relevant in clinical *S. aureus* biofilm-associated infections arising on medical devices or host tissues such as bone or heart valves, where bacterial attachment and biofilm initiation likely begin at low numbers.

### A platform for imaging biofilm and innate immune cell interactions *in vivo*

Despite decades of research on bacterial biofilms, many insights come from *in vitro* systems that may not capture the spatial or temporal complexity of *in vivo* infections (39). By applying intravital microscopy to a live foreign-body biofilm model, we directly visualized both biofilm architecture and host immune cell behavior in real time. Loss-of-function mutations in *icaADBC* abolished PNAG production within the biofilm matrix and accelerated bacterial clearance from the skin.

In wild-type infections, we observed a “dark zone” corresponding to a region with the PNAG-rich biofilm matrix. This region resembled the dead zone previously described in *Pseudomonas aeruginosa* biofilm infection in the cornea, which excludes both host and bacterial fluorescence (40). Although other components such as proteins or eDNA may also contribute to the dark zone, the absence of the dark zone in Δ*icaADBC*, as well as PgaB- and DspB-treated infections strongly supports a role for PNAG in forming this barrier. We note that PNAG was not directly visualized within the dark zone in the same imaging experiment shown in Fig. 6; we separately quantified PNAG abundance using the anti-PNAG antibody (Fig. 1D and E). We acknowledge that simultaneous imaging of PNAG alongside the dark zone phenotype, together with other ECM components such as proteins and eDNA, would more directly establish PNAG’s precise contribution to this structure. This remains an important avenue for future investigation.

Data from the Δ*rbf* mutant, which exhibits reduced *icaADBC* expression (35), further support the conclusion that mice fully clear Δ*rbf* and Δ*icaADBC* infections by 7 days post-infection. In contrast, the Δ*agr* strain, defective in quorum sensing, showed increased bacterial persistence at 7 days, consistent with Agr’s established role in biofilm dispersal and virulence regulation (36, 41). Together, these findings highlight how this *in vivo* imaging platform can be broadly applied to dissect the contributions of biofilm matrix components and their regulators, enabling mechanistic studies across strains, foreign-body surfaces, and infection models (3).

### PNAG limits neutrophil access and migration

Neutrophil elastase is a major protease released during neutrophil activation that degrades proteinaceous components of host and microbial extracellular matrices, potentially facilitating bacterial clearance (40). In our model, PNAG-deficient *S. aureus* was more accessible to neutrophils, resulting in enhanced bacterial clearance. We suggest that the PNAG-rich matrix likely restricts neutrophil elastase distribution, penetration, and/or enzymatic activity, limiting neutrophil-mediated killing.

Dynamic imaging revealed that neutrophil behavior differs markedly depending on PNAG presence. Neutrophils recruited to wild-type infection exhibited reduced motility, shorter track displacement, and limited penetration into the infection site. In contrast, neutrophils in Δ*rbf* and Δ*icaADBC* infections migrated more rapidly and deeply into the bacterial focus. This phenotype, most pronounced with the Δ*icaADBC* mutant, supports the idea that the PNAG matrix acts as a physical and biochemical barrier to immune cell infiltration. A recent study found that PNAG stimulates secretion of the chemokine CXCL10 from human PBMCs via a Dectin-1-Syk-CARD9 signaling axis, promoting the migration of CD14^+^CXCL10^+^ monocytes toward sites of infection (42). Whether this chemokine modulating activity plays a role concurrently with, or independently of, its role in neutrophil exclusion toward the biofilm matrix remains an open question. Integrating both immune signaling and biophysical barrier mechanisms represents an important direction for future work.

### Targeting biofilm integrity as a therapeutic strategy

The glycoside hydrolase PgaB, produced by the *pgaABCD* operon found in *Bordetella bronchiseptica* and *Escherichia coli* (43), contains dual deacetylase and hydrolase domains and is required for PNAG-dependent biofilm remodeling (24). In Staphylococcal biofilms, PgaB enzymatically cleaves PNAG, disrupting the exopolysaccharide matrix. Pathogens that do not produce PNAG are unaffected by its activity (44). PgaB is a highly specific, endo-acting, β-1,6-N-acetylglucosaminidase that exclusively targets partially deacetylated PNAG polymers (24). Because of its strict structural requirements for catalysis—including a β-1,6 glycosidic linkage, ≥1 deacetylated glucosamine (GlcN) residue in the –3 position relative to the cleavage site, and a minimal substrate length containing ≥4 consecutive residues (e.g., GlcN–GlcNAc–GlcNAc–GlcNAc) (24)—PgaB is not expected to act on host cell surface glycoconjugates. Rather, this study suggests that by degrading pathogen-derived PNAG, PgaB affects host immune function *in vivo*. In our model, PgaB treatment eliminated the dark zone, restored neutrophil access, and promoted bacterial clearance. Other PNAG-targeting enzymes, including dispersin B and other glycoside hydrolases from bacteria such as *P. aeruginosa* and *Aspergillus* species, have also shown therapeutic potential in *in vivo* infection models (45, 46). Our findings extend this work, illustrating that enzymatic disruption of PNAG can restore immune surveillance and bacterial clearance, suggesting that glycoside hydrolases may represent a viable adjunctive strategy for treating *S. aureus* biofilm infections. In parallel to enzymatic disruption, PNAG-targeted vaccination offers a complementary therapeutic avenue; recent work mapping the epitope specificity of a protective anti-PNAG monoclonal antibody enabled rational design of next-generation PNAG-conjugate vaccines that conferred near-complete protection against lethal *S. aureus* challenge in mice (47), underscoring PNAG as a multimodal target for both enzymatic and antibody-based interventions.

### Conclusions

A recent systematic survey of putative bacterial extracellular polysaccharide biosynthesis operons revealed that genomes from 288 bacterial species contain a putative gene locus that encodes for PIA/PNAG production (43). These data not only suggest an evolutionarily conserved origin and mechanism for PNAG-mediated biofilm formation but also point to PgaB as a broadly targeting therapeutic for chronic infections caused by many different bacterial species. This report demonstrates a role for *icaADBC*-linked *S. aureus* biofilm formation *in vivo* and makes it tempting to speculate that glycoside hydrolases could be used to target *S. aureus* biofilm infections in the clinical setting.

## MATERIALS AND METHODS

### Mice

Animal experiments were performed with adult male and female 7- to 8-week-old mice. All mice were housed under specific pathogen-free conditions and received sterilized rodent chow and water *ad libitum*. Mice infected for longer than 24 h were housed in a biohazard facility biosafety level 2. C57BL/6J mice were purchased from The Jackson Laboratory and bred in-house. Ly6G-cre/Ai14 (Catchup^IVM-red^) mice were a kind gift from Matthias Gunzer. We crossed Catchup^IVM-red^ mice to CX3CR1^gfp/gfp^ mice to generate Catchup^IVM-red^ CX3CR1^gfp/wt^ double reporter mice, as previously described (34).

### S. aureus

*S*. *aureus* strain USA400 MW2 (48) and its genetically engineered mutant strains were used for every experiment. MW2 wild-type and MW2 Δ*rbf* were kindly gifted to us from Dr. Chia Lee (UAMS). Bacteria were grown in brain heart infusion (BHI) broth medium at 37°C while shaking at 225 rpm. When required, bacteria were transformed with pCM29 to constitutively express GFP (49). For MW2-GFP growth, chloramphenicol (10 μg/mL) was added for plasmid selection. For infection, *S. aureus* strains were sub-cultured in BHI medium without antibiotics until late exponential phase (OD_660_, 1.5), washed once with sterile PBS, and resuspended in 1 mL PBS.

### Standard microbiological and molecular biology methods

All strains and plasmids are listed in Table S1, and primers are listed in Table S2. All basic microbiological and molecular procedures were executed according to standard protocols (50). DNA concentrations were measured using the A260/A280 method with a Nanodrop 2000 Spectrophotometer (Thermo Fisher Scientific). Protein concentrations were determined using the Pierce 660 nm Protein Assay Reagent (Thermo Fisher Scientific), which was calibrated using bovine serum albumin standards. Genomic DNA (gDNA) isolation, plasmid preparation, and DNA gel extraction were performed using nucleotide purification kits purchased from Qiagen or BioBasics. Phusion DNA polymerase and BP Clonase II were purchased from ThermoFisher Scientific. Oligonucleotide primers were purchased from Integrated DNA Technologies. Sanger sequencing was outsourced to UCDNA Services at the University of Calgary. Lysostaphin, anhydrotetracycline (ATC), chloramphenicol (CHL), and carbenicillin (CAR) were purchased from Sigma-Aldrich. Antibiotic stock solutions were corrected for activity, filter-sterilized, split into 0.5 mL aliquots, and stored at −70°C until used.

### Microbiological media and buffers

Ultrapure water was used in all buffers and media. It was prepared in-house using a Milli-Q Direct Water Purification System (Millipore-Sigma). Phosphate-buffered saline (PBS) was purchased as a 20× concentrate (Amresco). It was diluted as needed in ultrapure water and then sterilized using an autoclave. *Escherichia coli* and *S. aureus* strains were routinely propagated in lysogeny broth (LB). LB was made with 10 g tryptone, 5.0 g yeast extract, and 5.0 g NaCl per 1.0 L of ultrapure water. Semi-solid LB agar in petri dishes was prepared by adding 15.0 g/L bacteriological agar to LB prior to autoclaving. *S. aureus* was propagated, as required, in brain-heart infusion (BHI) broth, tryptic soy broth (TSB), or tryptic soy agar (TSA) purchased from BD Difco and prepared according to the manufacturer’s directions in ultrapure water. As required, antibiotics and/or ATC were added to the media for selection during genetic manipulations as follows: for *E. coli*, CAR at 50 µg/mL^−1^ and CHL at 25 µg/mL^−1^, and for *S. aureus* MW2, CHL at 10 µg/mL^−1^ and ATC at 0.50 µg/mL^−1^. All strains were stored at −70°C in LB containing 16.7% glycerol.

### Construction of allelic exchange vectors and *S. aureus* deletion mutants

An in-frame deletion mutation in *icaADBC* was engineered in *S. aureus* MW2 using established protocols for two-step allelic exchange (25), but with some modifications. Briefly, primer pairs oTER334/oTER345 and oTER338/oTER344 were used to amplify DNA regions upstream and downstream of *icaA* and *icaC*, respectively. These PCR products were gel purified and then fused together using established protocols (51) for splicing-by-overlap-extension (SOE) PCR with primers oTER334 and oTER344, which had been tailed with attB1 and attB2 sequences, respectively. The SOE-PCR product was gel purified and then recombined with pKOR1 using BP Clonase II. This reaction mixture was transformed into *E. coli* DH5α via electroporation, plated onto LB + CAR agar, and incubated overnight at 30°C. Clones bearing the desired insert were identified via established protocols for colony PCR with M13F and M13R primers (oJJH367 and oJJH368, respectively; Table S2) (51, 52). The resulting allelic exchange vector, pKOR1::Δ*icaABDC* (pTER139; Table S1), was verified by Sanger sequencing using M13F and M13R primers.

The pTER139 plasmid was transformed into *E. coli* DC10B, outgrown, selected, and purified using standard methods to ensure restriction modification system compatibility (53) with *S. aureus* MW2. Subsequently, 5 µg pTER139 was electroporated into *S. aureus* MW2 via established protocols (53). Merodiploids were selected on TSA + CHL after 24 h of incubation at 30°C. Subsequently, several propagations were necessary to cure the pKOR1-derived allelic exchange vector. Here, 2–3 colonies were picked from these plates and transferred to 5 mL TSB + CHL and incubated overnight at 30°C and 250 RPM; 5 µL of this culture was then transferred to 5 mL TSB + CHL and incubated for 24 h at 42°C and 250 RPM. This culture was serially diluted and spread onto TSA + CHL and incubated at 42°C. Merodiploid colonies were collected with a sterile swab and transferred to 5 mL TSB and incubated overnight at 37°C and 250 RPM, and then serially diluted and spread on TSA + ATC for counterselection. Colonies were picked from these plates and spotted on TSA and TSA + CHL. Colony PCR using phenol extraction of *S. aureus* gDNA (54) from CHL-sensitive colonies was used to identify the Δ*icaADBC* mutant using primers oTER423 and oTER382 (Table S2). The deletion mutation was confirmed via Sanger sequencing of this PCR product using the same primers, yielding *S. aureus* TER49 (Table S1).

### Construction of *ica*-complemented *S. aureus* mutants

A pALC2073 complementation vector was employed to construct an icaR-*icaADBC* complemented strain of *S. aureus* MW2 Δ*icaADBC*. Briefly, the DNA regions of *icaR* to *icaC* were amplified from *S. aureus* MW2 genomic DNA using primer pairs oRP76/oRP78, incorporating KpnI and SacI restriction sites in the forward and reverse primers, respectively. Subsequently, the resulting PCR products were electrophoresed, gel-purified, and digested with KpnI-HF (R3142 from NEB) and SacI-HF (R3156 from NEB) following the NEB double digestion protocol. The digested fragments were gel-purified and quantified using the DNA NanoDrop method and then subjected to ligation with the pALC2073 plasmid, which had been similarly digested with KpnI-HF and SacI-HF. The ligation was performed at a 3:1 ratio of insert to vector using T4 DNA ligase (M0202 from NEB), resulting in the formation of the complementation vector pALC2073::*icaR-icaADBC* (pRTS25). This construct was transformed into *E. coli* K12 IM01B (LMBP 9581), plated onto LB + AMP + anhydrotetracycline (250 ng/mL), and incubated overnight at 37°C. Clones carrying the desired insert were verified through sequencing of the extracted plasmid by the Plasmidsaurus sequencing service.

Afterwards, pRTS25 extracted from *E. coli* K12 IM01B was electroporated into *S. aureus* MW2 Δ*icaADBC* using established protocols (46), plated onto TSA + CHL + anhydrotetracycline (250 ng/mL) agar, and incubated overnight at 37°C. The resulting MW2 Δ*icaADBC* complemented strain (RTS41) was validated through plasmid extraction and PCR confirmation of the *icaR-icaADBC* fragment using primer pairs oRP79/oRP80. In parallel, the pALC2073 vector was introduced into *E. coli* K12 IM01B and was electroporated into *S. aureus* MW2 Δ*icaADBC* as outlined above, generating the vector control strain (RTS35).

### Semi-quantitative PNAG dot blots

Dot blots for PNAG were executed by protocols modified from those previously described by our group to measure *P. aeruginosa* PSL (52). Here, *S. aureus* strains were grown overnight on TSA at 37°C. Subsequently, single colonies were picked from the agar with a sterile loop, transferred to 2 mL TSB containing 0.125% (wt/vol) glucose, and incubated overnight at 37°C and 250 RPM. Cell pellets were collected from 1 mL of each culture by centrifugation (21,000 RCF for 2 min), and the supernatant was discarded. The cells were suspended in 250 µL of 0.5 M EDTA (pH 8.0) and boiled at 100°C for 60 min. These boiled suspensions were again centrifuged (21,000 RCF for 10 min), and 220 µL of the supernatant was transferred to a new tube. Protein concentrations were measured for each sample using the A280 NanoDrop method and a bovine serum albumin protein standard curve (Thermo Fisher Cat. 23208). Afterward, 10 µL of proteinase K was added to each sample, and the samples were incubated at 56°C for 30 min, followed by a 100°C incubation for 15 min to inactivate proteinase K. These samples were diluted to 3,000, 1,500, and 750 ng/µL and stored at −20ᵒC.

Frozen samples were thawed and then boiled at 100°C for 5 min. A 2 µL aliquot for each sample was spotted in technical triplicate onto nitrocellulose membranes. The membrane was air dried and then rinsed in Tris-buffered saline containing 0.5% (wt/vol) Tween-20 (TBS-T). Blots were blocked with TBS-T containing 5% (wt/vol) skim milk powder for 30 min, which were placed at room temperature on an orbital shaker at 100 RPM. Afterward, the blocking buffer was replaced with TBS-T + 5% (wt/vol) skim milk powder containing the human-α-PNAG antibody (32) at a 1:1,000 dilution. The blot was then incubated at room temperature on the orbital shaker for 1 h and then rinsed three times with TBS-T (3 × 10 min, on the orbital shaker). Subsequently, the blot was labeled with horseradish peroxidase (HRP)-conjugated goat anti-human IgG antibody (Invitrogen, catalog number 31410) using a 1:3,333 dilution in TBS-T. The blot was then incubated at room temperature on the orbital shaker for 1 h and then rinsed three times with TBS-T (3 × 10 min, on the orbital shaker). Subsequently, the blot was labeled with HRP-conjugated goat anti-human IgG antibody (Invitrogen, catalog number 31410) using a 1:3,333 dilution in TBS-T. The blot was again incubated at room temperature on the orbital shaker for 1 h and then rinsed three times with TBS-T as described above. Finally, the HRP-conjugated secondary antibody was visualized with Super Signal West Dura Extended Duration Substrate (Thermo Fisher Scientific) and imaged using the FluorChemQ gel documentation system (Proteinsimple). Images were captured and analyzed using Alphaview (v3.4.0) software (Proteinsimple). Analysis was executed in biological and technical triplicate. Each sample was run in technical triplicate. One biological replicate was run per blot; three biological replicates were tested.

### *In vitro* biofilm assay

*S. aureus* MW2 wild type or Δ*icaADBC* was grown in TSB medium containing 0.125% glucose for 24 h, and biofilms were stained with 0.1% crystal violet using two different *in vitro* biofilm assays: the Minimum Biofilm Eradication Concentration (MBEC) assay utilizing the Calgary Biofilm Device, as previously described (27, 28), and the microplate biofilm assay (29).

### Opsonophagocytic killing assay

Opsonophagocytic killing of *S. aureus* strains obtained from the collection of the Network on Antimicrobial Resistance in *S. aureus* (NARSA) was carried out as previously described (22). Briefly, 100 µL each of 10^7^/mL human PMNs, 25% rabbit Lo-tox complement, human IgG monoclonal antibody (MAb) to PNAG (32), or control human IgG1 MAb to *P. aeruginosa* alginate (55) were mixed together using MEM with 1% BSA as diluent for 90 min. Assay contents were diluted in tryptic soy broth containing 0.05% Tween 20 to disaggregate bacterial cells, and dilutions were plated on tryptic soy agar with 5% sheep blood. Colony-forming units were enumerated the next day, and the percentage of bacteria killed was calculated using the cfu counts in the tube with the control MAb as the denominator.

### *S. aureus* agar bead preparation

*S. aureus*-inoculated agar beads were used to model a foreign-body infection, as previously described (34). Bacteria were suspended in 1 mL PBS, adjusted to an OD_660_ of 0.100, and then diluted 10-fold; 250 μL of bacteria suspension was then added to 2.25 mL of freshly autoclaved, warm, liquid 1.5% BHI agar. The agar-bacterium mixture was slowly dropped into 40 mL ice-cold mineral oil solution containing 400 μL Tween 20 to prevent bead clumping. Gentle stirring for 15 min in an ice bath yielded spherical *S. aureus* agar beads. From there, beads were washed with sterile PBS by spinning in a centrifuge at 2,000 rpm for 10 min. This wash step was repeated up to eight times to remove mineral oil coating the beads. Beads were stored at 4°C for up to 2 days, and fresh beads were made before every experiment. Validation of *S. aureus* bead concentrations of every bead batch was confirmed by mechanical disruption three times with a 30-gauge insulin syringe in 1 mL sterile PBS; 50 μL of the bead solution was plated onto BHI agar plates and grown overnight at 37°C for enumeration of CFUs. The average of four beads determined the bead concentration of the batch.

### Infection model: *S. aureus* agar beads

For bead infections, mice were anesthetized with isoflurane gas, back hair was shaved, and hair was chemically removed with Nair hair removal cream. Nair cream was washed off with water, and the skin was dried with a gauze pad. Mice were tattooed with green animal tattoo ink to permanently mark the infection site. A single bead was picked up with forceps and placed onto the bore of an 18-gauge needle connected to a syringe containing 50 μL sterile PBS, and the bead was moved back by gentle pull of the syringe, which moved the bead into the needle tip. The bead was injected subcutaneously into the dorsal flank skin within the tattooed region. For CFU experiments, skin infections (1 cm^2^ biopsy of full thickness skin) were collected at different time points and homogenized in PBS; serial dilutions were plated on BHI agar plates, and colonies were counted after 18 h at 37°C.

### Scanning electron microscopy

Skin infections were harvested and immediately fixed in 3% glutaraldehyde and paraformaldehyde for 2 h. Samples were dehydrated in increasing concentrations of ethanol (30%, 50%, 70%, 80%, 90%, and 100%), 10 min for each wash. Samples were transferred to hexamethyldisilazane for 1 h and air-dried overnight. Samples were sputter coated with 10 nm platinum prior to imaging. SEM imaging was done on the XL30 30 kV scanning electron microscope.

### Skin imaging

Mice were anesthetized (ketamine [200 mg/kg] and xylazine [10 mg/kg] intraperitoneally), and a jugular catheter was inserted as previously described (34). Anesthetics were administered through the intravenous catheter at regular intervals to maintain the mouse in a surgical plane of anesthesia. The skin surgical procedure was performed as previously described (34). Briefly, mice were placed on a heating pad (World Precision Instruments) maintained at 37°C. A midline incision was made on the dorsal side of the mouse, and the dorsal side of the flank skin was exposed and secured using silk stay sutures onto a skin prep board (3D-printed in-house). A superfusion system was set up with Hanks’ balanced salt solution (HBSS) heated to 37°C to perfuse the exposed skin tissue at a flow rate of 0.05. A cover glass was placed on top of the exposed skin for imaging.

### Resonant scanning multiphoton intravital microscopy

Intravital image acquisition of the skin was performed with an upright Leica SP8 resonant scanning multiphoton microscope equipped with a 20×0.95 NA water objective. A tunable multiphoton laser was set to 940 nm for excitation of GFP, tdTomato, and qTracker655 for blood vessels. Second harmonic generation was visualized at an emission of 470 nm. External hybrid detectors were used to detect emission at 620–680 nm (HyD-RLD1), 565–620 nm (HyD-RLD2), 495–565 nm (HyD-RLD3), and <495 nm (HyD-RLD4). For 3D time-lapse videos, three fields of view were selected within the infection area at a 50 μm z-stack with a 5 μm z-step size. Intervals were set to 30.0 s, and videos were acquired over 20 min. After videos were acquired, 2–3 3D regions were imaged to capture the infection area at the following dimensions: 2 × 5 tile scan at 200 μm z-stack with 5 μm z-step size. All videos and images used a line averaging of 16.

### Image processing, analysis, and quantification

Raw imaging data were processed and quantified using Imaris Bitplane version 9.5. A Gaussian filter and background subtraction were applied to all images. A MATLAB XTension “Channel Arithmetics” was run to subtract the GFP signal from (Ch2[neutrophils]-Ch3[*S. aureus* GFP]). 3D surface models of collagen were generated in Imaris using default parameters. Imaris spot function was used for automated cell counting using default parameters, and then, all spots were filtered by volume to exclude spots <5 µm^3^. The total number of neutrophil spots would indicate the total neutrophil recruitment to the infection. For the quantification of neutrophil and monocyte infiltration, a 25 μm z-stack was cropped at the focal plane of the infection site, a mask was applied to the collagen surface, and then an intensity max filter was applied to neutrophil (tdTomato+) or monocyte (GFP+) spots as collagen mask + or collagen mask−. If neutrophils were identified as collagen mask−, then those neutrophils were quantified as infiltrated into the infection site. For the quantification of immune cell track length and velocity, tdTomato+ spots were tracked with Brownian motion over the 10-min video, and position XYZ coordinates were exported and added into RStudio where the data were quantified using an R script for the generation of the spider plots and velocity measurements. For the quantification of the dark zone in biofilm infections, a 2D region was cropped at the infection site, and the sum of collagen area, neutrophil area, and the GFP bacteria area was subtracted from the total region area. For overlapping areas, a mask was applied, and a new channel was duplicated such that any signal inside the mask was set to zero to ensure that the overlapping areas were not duplicated.

### Infection model: bloodstream *S. aureus* infection

Bacterial strains were grown in 3 mL BHI overnight at 37°C while shaking. Subcultures of 150 µL bacteria in 3 mL BHI were grown for 2 h at 37°C; 1 mL of subculture was spun down and resuspended in sterile saline and adjusted to 5e8 CFU/mL. Mice were injected intravenously with 200 µL of bacteria (1e8 CFU inoculum) of either *S. aureus* MW2 wild type, ∆*rbf*, or ∆*icaADBC*. Infected mice were regularly monitored for weight and illness behaviors. Euthanasia was performed when the humane endpoint was reached (20% weight loss) or clinical signs of severe systemic illness were observed.

### Flow cytometry

Skin biopsies of the infected area were harvested (1 cm^2^ section) and collected into cold HBSS. Skin tissue was digested as previously described (34). Skin tissue was incubated at 37°C with gentle rotation for 75 min in 2 mL of HBSS containing 3% FBS, 5 mM EDTA, and 0.8 mg/mL collagenase II (Worthington). Following enzymatic digestion, tissue was passed through a 70-μm filter and washed with HBSS containing 3% FBS and 5 mM EDTA. A debris removal step was performed as per the manufacturer’s protocol in the debris removal kit (Miltenyi Biotech). Single cells were resuspended in 800 μL of HBSS containing 3% FBS and 5 mM EDTA, and 200 μL was used for antibody staining. Blood was obtained by intra-cardiac collection from anesthetized mice, and 50 μL of blood lysed with ACK lysing buffer. Cells were first stained with Fc blocking antibody (1:200) and Ghost Red 710 fixable viability dye (1:6,400) in HBSS for 30 min on ice. CXCR4 antibody staining was done at 37°C for 20 min. Next, cell suspensions were stained for remaining surface antigens in HBSS supplemented with 3% FBS and 5 mM EDTA for 20 min on ice. After washing with HBSS containing 3% FBS and 5 mM EDTA, the cells were fixed with 1% paraformaldehyde in HBSS for 15 min on ice and then run the following day on the Cytek spectral cytometer. All flow cytometry experiments were analyzed with FlowJo v10 (Tree Star).

### Antibodies

Antibodies used for flow cytometry were purchased from eBioscience, BioLegend, or BD Biosciences. Dead cells were excluded by a fixable viability dye (Ghost Red 710, Tonbo Biosciences, 1:6,400). The antibodies in Table S4 were used for flow cytometry.

### Whole-mount 3D multiphoton microscopy

Skin samples were harvested from mice and fixed in 4% PFA for 48 h at 4°C. Samples were then washed 3 times in 1% PBS for 1 h at 4°C, shaking, and left in 1% BSA, 1% Triton X-100 PBS overnight at 4%, shaking. Tissues were then stained with rabbit anti-S100a9 antibody (Abcam, 1:500) and chicken anti-GFP antibody (AvesLabs, 1:200) in 1% Triton X-100 and 1% BSA PBS for 3 days at 4°C, shaking. Next, the samples were washed and stained with secondary antibodies (1:1,000) for 2 days at 4°C, shaking. The tissues were washed and mounted onto a glass coverslip and imaged using a Leica SP8 multiphoton microscope.

### *In vivo* enzyme treatment for biofilm disruption

A PgaB ortholog of *Bordetella bronchiseptica* (PgaB¬Bb) that only contained the C-terminus of PgaB was shown to have effective glycoside hydrolase activity comparable to other enzymes such as dispersin B (24), and this was the enzyme used in this work. The biofilm-disrupting enzyme, PgaB, or mutant, nonfunctional enzyme PgaB^D474N^ was injected subcutaneously into the mouse flank skin at the time of infection with the *S. aureus* bead. For PgaB and PgaB^D474N^, 0.4 mg/mouse was injected in a 50 μL volume.

### Statistical analysis and experimental design

In most experiments, sample size was determined based on previous studies within the lab using these techniques. For intravital microscopy, we were limited by imaging only one mouse at a time; hence, a minimum of 1 experimental mouse and one control mouse was imaged per day. Sample size was determined based on prior studies and literature using similar experimental paradigms. In instances where the approach had not previously been used, a minimum of four animals per group was utilized. All experiments were replicated at least once with similar findings, and all replications were successful. For all experiments that required either pharmacological treatment or different infection conditions, mice were randomized. The investigators were not blinded during experiments because treatments and data collection were performed by the same researcher. For image analysis, the images were randomly assigned a key by the researcher, and all images were processed using the same workflow; therefore, image analysis was blinded after data collection. Statistical analyses were performed using Prism 9 (GraphPad Software Inc., v9.1.1, La Jolla, CA). Statistical tests are described for each figure in the figure legend. A normality test was performed for all data to determine whether a parametric or non-parametric statistical test would be used. A *P*-value <0.05 was considered statistically significant.

## AFTER EPUB

*[This article was published on 31 July 2026 with a missing initial for Mario A. Vargas. The byline was corrected in the current version, posted on 6 August 2026.]*

## SUPPLEMENTAL MATERIAL

Supplemental Material mbio.00522-26-s0001.docx Figures S1-S5, Tables S1-S4, and Video S1 legend.

Video S1 mbio.00522-26-s0002.mp4 Neutrophil behavior during biofilm infection.

ASM does not own the copyrights to Supplemental Material that may be linked to, or accessed through, an article. The authors have granted ASM a non-exclusive, world-wide license to publish the Supplemental Material files. Please contact the corresponding author directly for reuse.

## References

[1] Collaborators GAR. 2022. Global mortality associated with 33 bacterial pathogens in 2019: a systematic analysis for the global burden of disease study 2019. Lancet 400:2221–2248. doi:10.1016/S0140-6736(22)02185-7 36423648 PMC9763654

[2] Murray CJL, Ikuta KS, Sharara F, Swetschinski L, Robles Aguilar G, Gray A, Han C, Bisignano C, Rao P, Wool E, et al.. 2022. Global burden of bacterial antimicrobial resistance in 2019: a systematic analysis. Lancet 399:629–655. doi:10.1016/S0140-6736(21)02724-0 35065702 PMC8841637

[3] Schilcher K, Horswill AR. 2020. Staphylococcal biofilm development: structure, regulation, and treatment strategies. Microbiol Mol Biol Rev 84:e00026-19. doi:10.1128/MMBR.00026-19 32792334 PMC7430342

[4] Costerton JW, Stewart PS, Greenberg EP. 1999. Bacterial biofilms: a common cause of persistent infections. Science 284:1318–1322. doi:10.1126/science.284.5418.1318 10334980

[5] Senthi S, Munro JT, Pitto RP. 2011. Infection in total hip replacement: meta-analysis. Int Orthop 35:253–260. doi:10.1007/s00264-010-1144-z 21085957 PMC3032119

[6] Karygianni L, Ren Z, Koo H, Thurnheer T. 2020. Biofilm matrixome: extracellular components in structured microbial communities. Trends Microbiol 28:668–681. doi:10.1016/j.tim.2020.03.016 32663461

[7] Moormeier DE, Bayles KW. 2017. *Staphylococcus aureus* biofilm: a complex developmental organism. Mol Microbiol 104:365–376. doi:10.1111/mmi.13634 28142193 PMC5397344

[8] Foster TJ, Geoghegan JA, Ganesh VK, Höök M. 2014. Adhesion, invasion and evasion: the many functions of the surface proteins of *Staphylococcus aureus*. Nat Rev Microbiol 12:49–62. doi:10.1038/nrmicro3161 24336184 PMC5708296

[9] Cheung GYC, Joo H-S, Chatterjee SS, Otto M. 2014. Phenol-soluble modulins--critical determinants of staphylococcal virulence. FEMS Microbiol Rev 38:698–719. doi:10.1111/1574-6976.12057 24372362 PMC4072763

[10] Lacey KA, Serpas L, Makita S, Wang Y, Rashidfarrokhi A, Soni C, Gonzalez S, Moreira A, Torres VJ, Reizis B. 2023. Secreted mammalian DNases protect against systemic bacterial infection by digesting biofilms. J Exp Med 220:e20221086. doi:10.1084/jem.20221086 36928522 PMC10037111

[11] Heilmann C, Schweitzer O, Gerke C, Vanittanakom N, Mack D, Götz F. 1996. Molecular basis of intercellular adhesion in the biofilm-forming *Staphylococcus epidermidis*. Mol Microbiol 20:1083–1091. doi:10.1111/j.1365-2958.1996.tb02548.x 8809760

[12] Joo H-S, Otto M. 2012. Molecular basis of *in vivo* biofilm formation by bacterial pathogens. Chem Biol 19:1503–1513. doi:10.1016/j.chembiol.2012.10.022 23261595 PMC3530155

[13] O’Gara JP. 2007. Ica and beyond: biofilm mechanisms and regulation in *Staphylococcus epidermidis* and *Staphylococcus aureus*. FEMS Microbiol Immunol 270:179–188. doi:10.1111/j.1574-6968.2007.00688.x 17419768

[14] Pokrovskaya V, Poloczek J, Little DJ, Griffiths H, Howell PL, Nitz M. 2013. Functional characterization of *Staphylococcus epidermidis* IcaB, a de-N-acetylase important for biofilm formation. Biochemistry 52:5463–5471. doi:10.1021/bi400836g 23866051

[15] Vuong C, Kocianova S, Voyich JM, Yao Y, Fischer ER, DeLeo FR, Otto M. 2004. A crucial role for exopolysaccharide modification in bacterial biofilm formation, immune evasion, and virulence. J Biol Chem 279:54881–54886. doi:10.1074/jbc.M411374200 15501828

[16] Kristian SA, Birkenstock TA, Sauder U, Mack D, Götz F, Landmann R. 2008. Biofilm formation induces C3a release and protects *Staphylococcus epidermidis* from IgG and complement deposition and from neutrophil-dependent killing. J Infect Dis 197:1028–1035. doi:10.1086/528992 18419540

[17] Yu L, Hisatsuclinical sne J, Kutsuno S, Sugai M. 2023. New molecular mechanism of superbiofilm elaboration in a *Staphylococcus aureus* clinical strain. Microbiol Spectr 11:e04425-22. doi:10.1128/spectrum.04425-22 36719203 PMC10100805

[18] Cramton SE, Gerke C, Schnell NF, Nichols WW, Götz F. 1999. The intercellular adhesion (ica) locus is present in *Staphylococcus aureus* and is required for biofilm formation. Infect Immun 67:5427–5433. doi:10.1128/IAI.67.10.5427-5433.1999 10496925 PMC96900

[19] Fitzpatrick F, Humphreys H, O’Gara JP. 2005. Evidence for icaADBC-independent biofilm development mechanism in methicillin-resistant *Staphylococcus aureus* clinical isolates. J Clin Microbiol 43:1973–1976. doi:10.1128/JCM.43.4.1973-1976.2005 15815035 PMC1081404

[20] Brooks JL, Jefferson KK. 2014. Phase variation of poly-N-acetylglucosamine expression in *Staphylococcus aureus*. PLoS Pathog 10:e1004292. doi:10.1371/journal.ppat.1004292 25077798 PMC4117637

[21] Toledo-Arana A, Merino N, Vergara-Irigaray M, Débarbouillé M, Penadés JR, Lasa I. 2005. *Staphylococcus aureus* develops an alternative, ica-independent biofilm in the absence of the arlRS two-component system. J Bacteriol 187:5318–5329. doi:10.1128/JB.187.15.5318-5329.2005 16030226 PMC1196035

[22] Kropec A, Maira-Litran T, Jefferson KK, Grout M, Cramton SE, Götz F, Goldmann DA, Pier GB. 2005. Poly-N-acetylglucosamine production in *Staphylococcus aureus* is essential for virulence in murine models of systemic infection. Infect Immun 73:6868–6876. doi:10.1128/IAI.73.10.6868-6876.2005 16177366 PMC1230935

[23] Lin MH, Shu JC, Lin LP, Chong KY, Cheng YW, Du JF, Liu S-T. 2015. Elucidating the crucial role of poly N-acetylglucosamine from *Staphylococcus aureus* in cellular adhesion and pathogenesis. PLoS One 10:e0124216. doi:10.1371/journal.pone.0124216 25876106 PMC4398431

[24] Little DJ, Pfoh R, Le Mauff F, Bamford NC, Notte C, Baker P, Guragain M, Robinson H, Pier GB, Nitz M, Deora R, Sheppard DC, Howell PL. 2018. PgaB orthologues contain a glycoside hydrolase domain that cleaves deacetylated poly-β(1,6)-N-acetylglucosamine and can disrupt bacterial biofilms. PLoS Pathog 14:e1006998. doi:10.1371/journal.ppat.1006998 29684093 PMC5933820

[25] Bae T, Schneewind O. 2006. Allelic replacement in *Staphylococcus aureus* with inducible counter-selection. Plasmid 55:58–63. doi:10.1016/j.plasmid.2005.05.005 16051359

[26] Croes S, Deurenberg RH, Boumans M-L, Beisser PS, Neef C, Stobberingh EE. 2009. *Staphylococcus aureus* biofilm formation at the physiologic glucose concentration depends on the *S. aureus* lineage. BMC Microbiol 9:229. doi:10.1186/1471-2180-9-229 19863820 PMC2774858

[27] Ceri H, Olson ME, Stremick C, Read RR, Morck D, Buret A. 1999. The calgary biofilm device: new technology for rapid determination of antibiotic susceptibilities of bacterial biofilms. J Clin Microbiol 37:1771–1776. doi:10.1128/JCM.37.6.1771-1776.1999 10325322 PMC84946

[28] Harrison JJ, Stremick CA, Turner RJ, Allan ND, Olson ME, Ceri H. 2010. Microtiter susceptibility testing of microbes growing on peg lids: a miniaturized biofilm model for high-throughput screening. Nat Protoc 5:1236–1254. doi:10.1038/nprot.2010.71 20595953

[29] O’Toole GA. 2011. Microtiter dish biofilm formation assay. J Vis Exp 47:2437. doi:10.3791/2437 PMC3182663 21307833

[30] Berglund ED, Li CY, Poffenberger G, Ayala JE, Fueger PT, Willis SE, Jewell MM, Powers AC, Wasserman DH. 2008. Glucose metabolism *in vivo* in four commonly used inbred mouse strains. Diabetes 57:1790–1799. doi:10.2337/db07-1615 18398139 PMC2453626

[31] McKenney D, Pouliot KL, Wang Y, Murthy V, Ulrich M, Döring G, Lee JC, Goldmann DA, Pier GB. 1999. Broadly protective vaccine for *Staphylococcus aureus* based on an *in vivo*-expressed antigen. Science 284:1523–1527. doi:10.1126/science.284.5419.1523 10348739

[32] Kelly-Quintos C, Cavacini LA, Posner MR, Goldmann D, Pier GB. 2006. Characterization of the opsonic and protective activity against *Staphylococcus aureus* of fully human monoclonal antibodies specific for the bacterial surface polysaccharide poly-N-acetylglucosamine. Infect Immun 74:2742–2750. doi:10.1128/IAI.74.5.2742-2750.2006 16622211 PMC1459728

[33] Atkins KL, Burman JD, Chamberlain ES, Cooper JE, Poutrel B, Bagby S, Jenkins ATA, Feil EJ, van den Elsen JMH. 2008. *S. aureus* IgG-binding proteins SpA and Sbi: host specificity and mechanisms of immune complex formation. Mol Immunol 45:1600–1611. doi:10.1016/j.molimm.2007.10.021 18061675

[34] Kratofil RM, Shim HB, Shim R, Lee WY, Labit E, Sinha S, Keenan CM, Surewaard BGJ, Noh JY, Sun Y, Sharkey KA, Mack M, Biernaskie J, Deniset JF, Kubes P. 2022. A monocyte-leptin-angiogenesis pathway critical for repair post-infection. Nature 609:166–173. doi:10.1038/s41586-022-05044-x 35948634

[35] Cue D, Lei MG, Luong TT, Kuechenmeister L, Dunman PM, O’Donnell S, Rowe S, O’Gara JP, Lee CY. 2009. Rbf promotes biofilm formation by *Staphylococcus aureus* via repression of *icaR*, a negative regulator of *icaADBC*. J Bacteriol 191:6363–6373. doi:10.1128/JB.00913-09 19684134 PMC2753044

[36] Kong K-F, Vuong C, Otto M. 2006. *Staphylococcus* quorum sensing in biofilm formation and infection. Int J Med Microbiol 296:133–139. doi:10.1016/j.ijmm.2006.01.042 16487744

[37] Zeng W, Song Y, Wang R, He R, Wang T. 2023. Neutrophil elastase: from mechanisms to therapeutic potential. J Pharm Anal 13:355–366. doi:10.1016/j.jpha.2022.12.003 37181292 PMC10173178

[38] Harding MG, Zhang K, Conly J, Kubes P. 2014. Neutrophil crawling in capillaries; a novel immune response to *Staphylococcus aureus*. PLoS Pathog 10:e1004379. doi:10.1371/journal.ppat.1004379 25299673 PMC4192594

[39] Bjarnsholt T, Whiteley M, Rumbaugh KP, Stewart PS, Jensen PØ, Frimodt-Møller N. 2022. The importance of understanding the infectious microenvironment. Lancet Infect Dis 22:e88–e92. doi:10.1016/S1473-3099(21)00122-5 34506737 PMC9190128

[40] Thanabalasuriar A, Scott BNV, Peiseler M, Willson ME, Zeng Z, Warrener P, Keller AE, Surewaard BGJ, Dozier EA, Korhonen JT, Cheng L-T, Gadjeva M, Stover CK, DiGiandomenico A, Kubes P. 2019. Neutrophil extracellular traps confine *Pseudomonas aeruginosa* ocular biofilms and restrict brain invasion. Cell Host Microbe 25:526–536. doi:10.1016/j.chom.2019.02.007 30930127 PMC7364305

[41] Vuong C, Saenz HL, Götz F, Otto M. 2000. Impact of the agr quorum-sensing system on adherence to polystyrene in *Staphylococcus aureus*. J Infect Dis 182:1688–1693. doi:10.1086/317606 11069241

[42] Gheitasi R, Weiss D, Müller MM, Sommer K, Röll D, Mosig A, Pletz MW, Makarewicz O. 2025. *Staphylococcus aureus* biofilm-associated component PNAG stimulates the secretion of the immunomodulatory chemokine CXCL10 via Dectin-1 signaling. Commun Biol 8:1136. doi:10.1038/s42003-025-08503-z 40745039 PMC12314102

[43] Bundalovic-Torma C, Whitfield GB, Marmont LS, Howell PL, Parkinson J. 2020. A systematic pipeline for classifying bacterial operons reveals the evolutionary landscape of biofilm machineries. PLoS Comput Biol 16:e1007721. doi:10.1371/journal.pcbi.1007721 32236097 PMC7112194

[44] Wang S, Breslawec AP, Alvarez E, Tyrlik M, Li C, Poulin MB. 2019. Differential recognition of deacetylated PNAG oligosaccharides by a biofilm degrading glycosidase. ACS Chem Biol 14:1998–2005. doi:10.1021/acschembio.9b00467 31430121

[45] Yu S, Su T, Wu H, Liu S, Wang D, Zhao T, Jin Z, Du W, Zhu M-J, Chua SL, Yang L, Zhu D, Gu L, Ma LZ. 2015. PslG, a self-produced glycosyl hydrolase, triggers biofilm disassembly by disrupting exopolysaccharide matrix. Cell Res 25:1352–1367. doi:10.1038/cr.2015.129 26611635 PMC4670989

[46] Snarr BD, Baker P, Bamford NC, Sato Y, Liu H, Lehoux M, Gravelat FN, Ostapska H, Baistrocchi SR, Cerone RP, Filler EE, Parsek MR, Filler SG, Howell PL, Sheppard DC. 2017. Microbial glycoside hydrolases as antibiofilm agents with cross-kingdom activity. Proc Natl Acad Sci USA 114:7124–7129. doi:10.1073/pnas.1702798114 28634301 PMC5502622

[47] Tan Z, Yang W, O’Brien NA, Pan X, Ramadan S, Marsh T, Hammer N, Cywes-Bentley C, Vinacur M, Pier GB, Gildersleeve JC, Huang X. 2024. A comprehensive synthetic library of poly-N-acetyl glucosamines enabled vaccine against lethal challenges of *Staphylococcus aureus*. Nat Commun 15:3420. doi:10.1038/s41467-024-47457-4 38658531 PMC11043332

[48] Baba T, Takeuchi F, Kuroda M, Yuzawa H, Aoki K, Oguchi A, Nagai Y, Iwama N, Asano K, Naimi T, Kuroda H, Cui L, Yamamoto K, Hiramatsu K. 2002. Genome and virulence determinants of high virulence community-acquired MRSA. Lancet 359:1819–1827. doi:10.1016/s0140-6736(02)08713-5 12044378

[49] Pang YY, Schwartz J, Thoendel M, Ackermann LW, Horswill AR, Nauseef WM. 2010. Agr-Dependent interactions of *Staphylococcus aureus* USA300 with human polymorphonuclear neutrophils. J Innate Immun 2:546–559. doi:10.1159/000319855 20829608 PMC2982852

[50] Green MR, Sambrook J. 2012. Molecular cloning: a laboratory manual. Cold Spring Harbor, New York.

[51] Hmelo LR, Borlee BR, Almblad H, Love ME, Randall TE, Tseng BS, Lin C, Irie Y, Storek KM, Yang JJ, Siehnel RJ, Howell PL, Singh PK, Tolker-Nielsen T, Parsek MR, Schweizer HP, Harrison JJ. 2015. Precision-engineering the *Pseudomonas aeruginosa* genome with two-step allelic exchange. Nat Protoc 10:1820–1841. doi:10.1038/nprot.2015.115 26492139 PMC4862005

[52] Harrison JJ, Almblad H, Irie Y, Wolter DJ, Eggleston HC, Randall TE, Kitzman JO, Stackhouse B, Emerson JC, Mcnamara S, Larsen TJ, Shendure J, Hoffman LR, Wozniak DJ, Parsek MR. 2020. Elevated exopolysaccharide levels in *Pseudomonas aeruginosa* flagellar mutants have implications for biofilm growth and chronic infections. PLoS Genet 16:e1008848. doi:10.1371/journal.pgen.1008848 32530919 PMC7314104

[53] Monk IR, Shah IM, Xu M, Tan M-W, Foster TJ. 2012. Transforming the untransformable: application of direct transformation to manipulate genetically *Staphylococcus aureus* and *Staphylococcus epidermidis*. mBio 3:e00277-11. doi:10.1128/mBio.00277-11 22434850 PMC3312211

[54] Papakyriacou H, Vaz D, Simor A, Louie M, McGavin MJ. 2000. Molecular analysis of the accessory gene regulator (agr) locus and balance of virulence factor expression in epidemic methicillin-resistant *Staphylococcus aureus*. J Infect Dis 181:990–1000. doi:10.1086/315342 10720522

[55] Pier GB, Boyer D, Preston M, Coleman FT, Llosa N, Mueschenborn-Koglin S, Theilacker C, Goldenberg H, Uchin J, Priebe GP, Grout M, Posner M, Cavacini L. 2004. Human monoclonal antibodies to *Pseudomonas aeruginosa* alginate that protect against infection by both mucoid and nonmucoid strains. J Immunol 173:5671–5678. doi:10.4049/jimmunol.173.9.5671 15494518

