# Supplementary material for: Imaging Staphylococcus aureus biofilms in vivo reveals neutrophil exclusion by PNAG exopolysaccharide
Source: mBio. 2026 Jul 31;17(9):e00522-26. doi: 10.1128/mbio.00522-26 (PMC13556241; doi:10.1128/mbio.00522-26)
Supplement: Supplementary file 1 [file mbio.00522-26-s0001.docx]

**Supplemental material**

**Figure S1. PNAG expression is widespread and *ica*-dependent across diverse *S. aureus* strain backgrounds.** **(A)** Quantification of semi-quantitative PNAG dot blot for MW2, Δ*icaA and* Δ*icaC* strains. **(B)** Clinical isolates were incubated with the indicated concentrations of anti-PNAG IgG1, human PMNs, and rabbit complement. Bacterial survival was quantified after 90 min at 37 °C, and percent killing was calculated relative to an isotype-matched IgG1 control antibody specific for *P. aeruginosa* alginate. The y-axis lists strain types and sources of infection. MRSA, methicillin-resistant *S. aureus*; GISA, glycopeptide-insensitive *S. aureus*; BSI, bloodstream infection; PVL, Panton–Valentine leukocidin. **(C-D)** Quantification of semi-quantitative PNAG dot blot for *S. aureus* strains containing precisely engineered deletions in different *S. aureus* isolates **(C)** or *icaADBC* deletion in Newman ∆*spa*∆*sbi* **(D)**. **(E)** Quantification of mean PNAG signal within the skin infection with wild type or ∆*icaADBC* *S. aureus* bead at 24 hrs. Data are mean with SD **(A-D)**, mean with SEM **(E)**. Brown-Forsythe ANOVA test with Dunnett's T3 multiple comparisons test **(A, C, D)** were used. **p* < 0.05, ***p* < 0.01, ****p* < 0.001, *****p* < 0.0001.

**Figure S2. Effects on bacterial dissemination and systemic infection with PNAG-deficient strains. (A)** C57 mice were infected with *S. aureus* bead and peripheral organs were harvested at day 7 post-infection for quantification of CFUs **(B-C)** C57 mice were infected with 1e8 CFU *S. aureus* wild type, Δ*rbf*, or Δ*icaADBC* i.v. and mouse survival **(B)** and body weight **(C)** were recorded. *n* = 5 per group from 1 independent experiment.

**Figure S3. Neutrophil heterogeneity after *S. aureus* biofilm infection. (A-F)** C57 mice were infected *S. aureus* wild type, Δ*rbf,* and Δ*icaADCB* bead and spectral flow cytometry was performed at 24 h post-infection. **(A)** Gating strategy to identify neutrophils and monocytes. **(B-C)** Quantification of total numbers of neutrophils **(B)** and monocytes **(C)** at 24 h post-infection. **(D)** List of neutrophil markers used to characterize neutrophil heterogeneity at 24 h post-infection. Quantification of frequency **(E)** and MFI **(F)** of neutrophil subsets. *n =* 3-4 from one independent experiment. **(B-C, E-F)** One-way ANOVA with Tukey’s multiple comparisons test were used.

**Figure S4.** (**A-B**) C57 or NE-/- mice were infected with GFP-expressing *S. aureus* wild type and skin tissue was processed for whole mount immunofluorescence staining and imaged on the multiphoton microscope. (**A**) Representative 2D images at 24 h post-infection. (**B**) Quantification of percent neutrophil infiltration. n = 3-4 from 2 independent experiments. Data are mean ± SEM with Student t-test (**B**). **p < 0.01.

**Figure S5. Multiphoton intravital microscopy** **of infections with *S. aureus* Newman strains.** **A-B**) Catchup^ivm-red^ mice were infected with GFP-expressing *S. aureus* Newman wild type (**A**) or Newman Δ*icaADBC* (**B**) and imaged at 24 hours post infection. In these representative images, the red outline indicates the infection area. (**C**) Quantification of aggregate size vs number in wild type, Δ*rbf,* and Δ*icaADBC* bacteria within the infection site at 24 hrs. Each dot represents one mouse.

**Supplementary videos**

**Video S1. Neutrophil behaviour during biofilm infection.** Catchup^ivm-red^ mice were infected with GFP-expressing *S. aureus* MW2 WT, Δ*icaADBC,* or Δ*rbf* bead and imaged at 24h post-infection. A 3D timelapse video was taken and neutrophil behaviour was analyzed using Imaris. Video shows neutrophils (magenta), *S. aureus* GFP (yellow) and collagen (grey). The overlaid cell trajectory tracings and the grey spheres are neutrophil tracks from Imaris.

**Supplementary Tables**

**Table S1.** Bacterial strains used in this study.

| **Strain** | **Genotype, description or relevant characteristics** ^a^ | **Source** |
| --- | --- | --- |
| *Staphylococcus aureus* | | |
| MW2 | USA400 MW2, wild type | (34) |
| TER49 | MW2 Δ*icaADBC* | This study |
| RTS41 | TER49 complemented *icaADBC,* CHL^r^ | This study |
| RTS35 | TER49 vector control, CHL^r^ | This study |
| RTS74 | MW2 Δ*rbf* | (34) |
| Newman (CP5) | Newman type 5 capsular polysaccharide serotype, wild type | Dr. Gerald B. Pier |
| RTS78 | Newman (CP5) *ΔicaADBC* | Dr. Gerald B. Pier |
| RTS79 | RTS78 complemented *icaADBC* | Dr. Gerald B. Pier |
| MN8 (CP8) | MN8 type 8 capsular polysaccharide serotype, wild type | Dr. Gerald B. Pier |
| RTS81 | MN8(CP8) Δ*icaADBC* | Dr. Gerald B. Pier |
| RTS82 | RTS81 complemented *icaADBC* | Dr. Gerald B. Pier |
| NCTC 10833 | No capsule, wild type | Dr. Gerald B. Pier |
| RTS84 | NCTC 10833 Δ*icaADBC* | Dr. Gerald B. Pier |
| RTS85 | RTS85 complemented *icaADBC* | Dr. Gerald B. Pier |
| USA300 | USA300-JE2, wild type | Dr. Gerald B. Pier |
| RTS87 | USA300-JE2 Δ*icaADBC* | Dr. Gerald B. Pier |
| VJT 49.37 | Newman Δ*spa*Δ*sbi* | Dr. Victor Torres |
| RTS314 | Newman Δ*spa*Δ*sbi*Δ*icaADBC* | This study |
| *Escherichia coli* | | |
| K12 IM01B | Plasmids passage for high efficiency transformation of *S. aureus* MW2 | BCCM (Belgian Coordinated Collections of Microorganisms) |
| DH5α (NEB5α) | Strain for cloning | New England Biolabs |

^a^Abbreviations for antibiotic selection: CHL, chloramphenicol.

**Table S2.** Plasmids used in this study.

| **Plasmid** | **Genotype, description, or relevant characteristics** ^a^ | **Source** |
| --- | --- | --- |
| **Allelic exchange and cloning vectors** | | |
| pKOR1 | allelic exchange vector, AMP^r^, CHL^r^ | (25) |
| pTER139 | pKOR1*::*Δ*icaABDC*, AMP^r^ | This study |
| pCM29 | sGFP expression vector, AMP^r^, CHL^r^ | (47) |
| **Complementation vectors** | | |
| pALC2073 | complementation vector, anhydrotetracycline inducible (250ng/mL), AMP^r^, CHL^r^ | David Heinrichs (55) |
| pRTS25 | pALC2073::*icaR-icaADBC*, AMP^r^, CHL^r^ | This study |

^a^Abbreviations for antibiotic selection: AMP, ampicillin; CHL, chloramphenicol.

**Table S3.** Primers used in this study.

| **Oligonucleotide** | **DNA sequence**^a^ |
| --- | --- |
| **DNA sequencing** | |
| oTER334 | GGG G**AC AAG TTT GTA CAA AAA AGC AGG CT**T AGT TCG ATT TAT TTT GAT TCA TC |
| oTER345 | *CGT GTT TTT AAC ATA GCT TAA TAA TTA ATA AGC ATT* CAA AAA GTT AAA AAA TTG CAA TTT C |
| oTER338 | *AAT GCT TAT TAA TTA TTA AGC TA* |
| oTER344 | GGG G**AC CAC TTT GTA CAA GAA AGC TGG G**GT GAA AAC TGA AAA GCT G |
| oJJH367 (M13F) | GTA AAA CGA CGG CCA G |
| oJJH368 (M13R) | CAG GAA ACA GCT ATG AC |
| oTER423 | CAG AAA ATT CCT CAG GCG |
| oTER382 | CTG ATA CAG TGC GCA CAA GAG |
| oRTS76 | TTT TT**G GTA CC**T TAT TTC TTC AAA AAT ATA TTT AGT AGC GAA TAC ACT TCA TC |
| oRTS78 | CCC CC**G AGC TC**T TAA TAA GCA TTA ATG TTC AAT TTA TAT GGT TGT TTT CC |
| oRTS79 | CCT CCT TTT TGT TGA CAC TCT ATC |
| oRTS80 | CAG GGT TTT CCC AGT CAC GAC |

^a^*Italics* denote a region of complementarity for SOE-PCR; underlined sequence denotes a region specific to the targeted DNA amplicon; **bold** denotes an attachment (*att*) or restriction site for cloning.

**Table S4.** Antibodies used in this study.

| **Antibodies** | **Source** | **Identifier** |
| --- | --- | --- |
| Anti-mouse CD45 BV510 (clone 30-F11) | BioLegend | Cat# 103138 |
| Anti-mouse Ly6G BV650 (clone 1A8) | BD | Cat# 740554 |
| Anti-mouse CD11b BV750 (clone M1/70) | BioLegend | Cat# 101267 |
| Anti-mouse Ly6C PerCP Cy5.5 (clone HK1.4) | BioLegend | Cat# 129012 |
| Anti-mouse CD64 Alexa Fluor 647 (clone X54-5/7.1) | BD | Cat# 558539 |
| Anti-mouse CD182 (CXCR2) BUV563 (clone V48-2310) | BD | Cat# 748681 |
| Anti-mouse CD184 (CXCR4) BUV615 (clone 2B11) | BD | Cat# 752552 |
| Anti-mouse Siglec-F BUV737 (clone 1RNM44N) | BD | Cat# 750024 |
| Anti-mouse CD117 APC (clone 2B8) | BioLegend | Cat# 105812 |
| Anti-mouse CD49d FITC (clone R1-2) | BioLegend | Cat# 103606 |
| Anti-mouse CD62L BV605 (clone C068C2) | BioLegend | Cat# 104438 |
| Anti-mouse CD54 PE (clone YN1/1.7.4) | BioLegend | Cat# 116108 |
| Anti-mouse CD101 PE-Cy7 (clone Moushi101) | Invitrogen | Cat# 12-1011-82 |
